# Association Analysis of Genomic Loci Important for Grain Weight Control in Elite Common Wheat Varieties Cultivated with Variable Water and Fertiliser Supply

**DOI:** 10.1371/journal.pone.0057853

**Published:** 2013-03-04

**Authors:** Kunpu Zhang, Junjun Wang, Liyi Zhang, Chaowu Rong, Fengwu Zhao, Tao Peng, Huimin Li, Dongmei Cheng, Xin Liu, Huanju Qin, Aimin Zhang, Yiping Tong, Daowen Wang

**Affiliations:** 1 The State Key Laboratory of Plant Cell and Chromosome Engineering, Institute of Genetics and Developmental Biology, Chinese Academy of Sciences, Beijing, China; 2 Graduate University of Chinese Academy of Sciences, Beijing, China; 3 Guizhou Institute of Upland Crops, Guiyang, China; 4 Dry-Land Farming Institute, Hebei Academy of Agricultural and Forestry Sciences, Hengshui, China; 5 Jiyuan Institute of Agricultural Sciences, Jiyuan, China; Kansas State University, United States of America

## Abstract

Grain weight, an essential yield component, is under strong genetic control and markedly influenced by the environment. Here, by genome-wide association analysis with a panel of 94 elite common wheat varieties, 37 loci were found significantly associated with thousand-grain weight (TGW) in one or more environments differing in water and fertiliser levels. Five loci were stably associated with TGW under all 12 environments examined. Their elite alleles had positive effects on TGW. Four, two, three, and two loci were consistently associated with TGW in the irrigated and fertilised (IF), rainfed (RF), reduced nitrogen (RN), and reduced phosphorus (RP) environments. The elite alleles of the IF-specific loci enhanced TGW under well-resourced conditions, whereas those of the RF-, RN-, or RP-specific loci conferred tolerance to the TGW decrease when irrigation, nitrogen, or phosphorus were reduced. Moreover, the elite alleles of the environment-independent and -specific loci often acted additively to enhance TGW. Four additional loci were found associated with TGW in specific locations, one of which was shown to contribute to the TGW difference between two experimental sites. Further analysis of 14 associated loci revealed that nine affected both grain length and width, whereas the remaining loci influenced either grain length or width, indicating that these loci control grain weight by regulating kernel size. Finally, the elite allele of *Xpsp3152* frequently co-segregated with the larger grain haplotype of *TaGW2-6A*, suggesting probable genetic and functional linkages between *Xpsp3152* and *GW2* that are important for grain weight control in cereal plants. Our study provides new knowledge on TGW control in elite common wheat lines, which may aid the improvement of wheat grain weight trait in further research.

## Introduction

Common wheat (*Triticum aestivum* L.) is one of the most important staple food crops in the world [1]. Grain weight, commonly defined as the weight of 1000 grains (TGW), is an important component of wheat grain yield, and has a favourable effect on flour yield [2], [3]. Consequently, large grain has been a key trait selected during wheat domestication and modern wheat breeding [4], [5], and understanding and improving grain weight is an important area of wheat genetic and breeding studies [6]–[8].

Grain weight in cereal crops is a complex quantitative trait, which is negatively affected by environmental stresses but is positively enhanced by appropriate applications of nitrogen and phosphorus fertilisers [9]–[11]. In common wheat, a large number of quantitative trait loci (QTLs) for grain weight have been identified under irrigated and fertilised conditions (e.g., [3], [12]–[19]). In addition, many QTLs affecting grain weight under drought or rainfed conditions have been reported (e.g., [20]–[26]). QTLs influencing grain weight in environments with reduced supplies of nitrogen (N) or phosphorus (P) fertilisers have also been described (e.g., [20], [21], [27]–[33]). Because of the close correlation between grain weight and size, a number of studies have also been conducted for finding the QTLs affecting wheat grain size parameters, such as grain length and width under normal growth conditions (e.g., [3], [32]–[34]). Genetic control of these grain size component traits is also complex, each involving the participation of multiple QTLs. Nevertheless, several meta-QTLs that significantly influence grain length, width, and weight in different mapping populations have been identified in common wheat [35].

Although the available QTL data have contributed substantially to our current understanding of the genetic bases controlling grain weight and size, these QTLs have generally been identified in biparental segregating populations. QTL mapping in such populations is subject to low allele numbers and limited recombination [36]. Consequently, the QTLs revealed so far for grain weight and size in wheat may represent only parts of the genetic system underlying the control of these key yield component traits.

In contrast to QTL mapping using bi-parental populations, genome-wide association (GWA) analysis is a recently developed, high-resolution method for genetic mapping using existing germplasm (such as landraces, elite cultivars, and advanced breeding lines) based on linkage disequilibrium (LD) [36]–[40]. GWA analysis permits a survey of a wide range of alleles at each locus, detection of marker-trait associations at the whole genome level, and identification of elite alleles for significantly associated loci. Although elevated LD, which is inherent for primarily inbreeding crop species (such as rice and wheat), may increase the chance of false positives in GWA analysis, such errors may be minimised using a stringent statistical model that controls for both structure and kinship in the mapping population [38], [39], [41]. Consequently, GWA mapping is becoming increasingly important for identifying the genetic components controlling agronomic traits in cereal crops such as rice, barley, common wheat, durum wheat, and maize (e.g., [33], [42]–[47]).

In rice and maize for which the complete genome sequence is determined, GWA mapping is often facilitated by single nucleotide polymorphism (SNP) markers with known physical locations in the genome, and can lead to the discovery of major genes controlling the traits under investigation (e.g., [44], [47]). In barley and durum and common wheats, for which complete genome sequences are still unavailable, GWA analysis is commonly assisted by genetic markers, such as those based on simple sequence repeat (SSR), or those derived from diversity arrays technology (DArT). Such analysis may result in the identification of chromosomal loci linked to the target traits. Association analysis of grain weight and size in common wheat grown under well-resourced conditions has been reported in several studies [8], [33], [46], [48]–[51]. The chromosomal locations of many of the associated loci coincide with those of known QTLs involved in grain weight control. However, GWA mapping has also revealed new loci that have not been identified in past QTL investigations [8], [46], demonstrating that this approach has the potential to provide a more comprehensive understanding of the genetic determinants of grain weight control. To date, GWA analyses of grain weight under conditions of reduced N or P supply have not been reported in common wheat, although association studies of several drought-adaptive traits including grain weight have recently been described in durum and common wheat varieties [45], [51].

With the adoption of modern varieties and agronomic practices, China has recently become the largest wheat producer in the world, with a cultivation area of 23.6 million hectares and a total grain harvest of 112 million tonnes in 2008 [52], [53]. The wheat cultivation area in China is divided into 10 ecological zones, with zone II (i.e., facultative wheat zone in the Yellow and Huai river valleys) being most productive (accounting for about 65% of China’s total wheat acreage and production) [52]. Genetic improvement in yield-related traits (effective tiller number, grain number per spike, and TGW) has played important roles in increasing wheat production in China, with a mean 2.19 g increase in TGW per decade from the 1940s to the 2000s [8]. Additionally, irrigation and N and P fertiliser applications have aided the rise in wheat yield during this period [53], [54]. However, in the last ten years, the trends of yield increase in cereals (including wheat) have slowed, and the problems brought about by heavy use of irrigation and chemical fertilisers during crop growth (such as depletion of underground water resources, pollution of agro-ecosystems, and rising production costs) have raised serious concerns [53], [54]. The problems faced by China are shared in many regions worldwide [6], [55]. To contribute to clarify the genetic mechanisms behind grain weight control and to aid the efforts of developing water- and nutrient-efficient wheat varieties, we embarked on a systematic GWA analysis of yield-related traits under well-resourced or resource-limiting conditions using a panel of 94 elite wheat varieties cultivated primarily in region II. The main objectives of the work described here were to identify and analyse the genomic loci involved in TGW control under four different cultivation conditions using GWA mapping. The new insights obtained and their implications for further improving the wheat grain weight trait for efficient utilisation of water and N and P fertiliser resources are discussed.

## Materials and Methods

### Ethics Statement

We obtained the relevant permissions to grow the association mapping population (94 elite common wheat varieties) in the field during the wheat crop cycles in 08/09 and 09/10 in Hengshui (HS, 37°73′ N and 115° 72′ E, Hebei Province) and 09/10 in Jiyuan (JY, 35°08′ N and 112°57′ E, Henan Province) from the Dry-Land Farming Institute, Hebei Academy of Agricultural and Forestry Sciences and Jiyuan Institute of Agricultural Sciences, respectively. The study areas are not privately-owned or protected in any way. The field studies did not involve endangered or protected species.

### Plant Materials

A set of 94 elite common wheat varieties, released largely from the 1980s to 2000s, was used for the GWA analysis. Most of these varieties (89 lines) came from the facultative wheat zone in the Yellow and Huai river valleys (zone II), with the remaining five from the neighboring Northern winter wheat zone (zone I) (Figure S1). These varieties are generally winter hardy and have similar heading dates and flowering times. Zone II mainly includes the wheat cultivation regions in Henan and Shandong provinces and the southern part of Hebei province. Zone I encompasses the wheat cultivation regions in Beijing, Tianjin and the northern part of Hebei.

### Field Trials and TGW Evaluation

Field trials were conducted during the wheat crop cycles in 08/09 and 09/10 in HS and 09/10 in JY. The experiment followed a completely randomised block design with three replications at each location under four cultivation treatments: irrigated and fertilised (IF), rainfed (RF), reduced nitrogen (RN), and reduced phosphorus (RP). The levels of irrigation and nitrogen and phosphorus fertilisers applied to the four cultivation treatments are listed in Table S1. Each replication consisted of three rows of wheat plants; each row was 2 m in length, and the distance between adjacent rows was 20 cm. For each replication, TGW was measured using three separate samples, each containing 1,000 grains. Grain length and width measurements were performed on 200–250 grains per variety using a MARVIN grain analyser (Oftringen, Switzerland).

### DNA Extraction

DNA was extracted from young leaf tissues of each variety using the protocol recommended by Triticarte Pty. Ltd (http://www.triticarte.com.au). DNA quality was checked by electrophoresis on 0.8% agarose gels, and DNA concentration was determined with a NanoDropND-1000 UV-Vis spectrophotometer (NanoDrop Technologies, Wilmington, USA).

### Microsatellite Analysis

In total, 426 SSR primer pairs were selected to genotype the association mapping population. They included 109 BARC (*Xbarc*) [56], 21 CFA and 57 CFD (*Xcfa* and *Xcfd*) [57], 113 WMC (*Xwmc*) [58], 6 GDM (*Xgdm*) [59], 2 CFP (*Xcfp*) [60], 101 WMS [61], 17 PSP (*Xpsp*) [62], and 2 GPW (*Xgpw*, http://wheat.pw.usda.gov/ggpages/SSRclub/) primer sets. These primer sets were chosen because their target loci collectively showed a fairly even distribution among the 21 common wheat chromosomes. An ABI 3730 Analyser (Applied Biosystems, Foster City, CA, USA) was used to capture the amplification products by a fluorescence detection system for microsatellite markers. Fragment size was evaluated using GeneMapper v3.7 software (Applied Biosystems).

### DArT Genotyping

Ninety-four DNA samples were genotyped by DArT markers. DArT fingerprinting was performed at Diversity Arrays Technology Pty Limited (http://triticarte.com.au). About 7,000 polymorphic DArT markers were employed for scanning the 94 genotypes. Polymorphism was scored as described previously [63].

### Genetic Linkage Map

The SSR and DArT markers employed for the GWA analysis were integrated into a composite map based on previously reported maps from diverse genetic populations. They included (1) the microsatellite consensus map [64], (2) the composite wheat map (http//wheat.pw.usda.gov), (3) the Cranbrook × Halberd map (339 DArT loci) [65], (4) the Arina × NK93604 map (189 DArT loci) [66], (5) the Avocet × Saar map (112 DArT loci) [67], (6) the Colosseo × Lloyd map (392 DArT loci) [68], (7) wheat chromosome 3B physical and consensus maps [60], [69], and (8) the selected linkage groups of markers from nine different populations archived on the *Triticarte* website (http://www.triticarte.com.au). For mapping the relative genetic positions of *TaGW2-6A*, *Xpsp3152*, and *Xpsp3071* on chromosome 6A, the polymorphisms of these markers in the 168 double haploid lines, derived from the Huapei 3 × Yumai 57 cross [70], were scored, with the resultant data analysed by MAPMAKER/Exp version 3.0b [71]. The map positions of the three markers, along with those of previously published markers on 6A [70], were visualised with Mapchart version 2.1 software [72].

### Data Analyses

The mean values of the TGW and standard errors for the 12 environments were analysed using SPSS for Windows 13.0 (SPSS, http://www.spss.com) with a 95% confidence interval. The mean TGW for each treatment (TMTGW) was estimated using the best linear unbiased predictor method [73]. Broad sense heritability was calculated as described previously [74]. Population structure analysis for the 94 elite wheat accessions was performed using the NTSYSpc program [75] and STRUCTURE v2.2 software [76] based on the genotyping data obtained with 1,129 SSR and DArT markers distributed on 21 common wheat chromosomes. A relative kinship matrix was obtained by running SPAGeDi [77]. The basic genetic statistics, including total number of alleles and polymorphism information content (PIC) at each SSR or DArT locus, were calculated with the PowerMarker program v3.25 [78]. The statistical analysis of grain length and width data was carried out using SPSS for Windows 13.0.

Linkage disequilibrium (LD) between 1,129 unlinked markers, including pairwise estimates of the squared allele-frequency correlation (*r* ^2^) and the significance of each pair of loci, was estimated by TASSEL 2.1 (http://www.maizegenetics.net/). During LD estimation, SSR and DArT datasets were filtered for rare alleles with frequencies of less than 5%. The various statistical models in TASSEL 2.1 software (http://www.maizegenetics.net/) were evaluated for genome-wide marker-trait associations, with the mixed linear model (MLM) finally adopted. Both population structure (Q) and kinship (K) were taken into account during the marker-trait association analysis with MLM. The different sets of data from the 12 environments were each analysed for marker-trait associations. The *R^2^* value (percentage of variance explained) and the phenotypic effect on TGW (g) were both computed for the associated markers (loci).

Marker allele-assisted genotyping was conducted to compare the allelic effects of the associated loci on TGW, as described previously [8], [33]. Briefly, the 94 varieties were sorted into two groups according to the particular alleles that the individual genotypes carried for the locus under investigation. The average TGWs of the two groups, carrying the elite or inferior alleles of the concerned locus, were then calculated and compared. To improve reliability during the analysis of allelic effects, genotyping was conducted for only the associated loci whose elite alleles were present in more than 20 members of the 94 varieties. For the associated loci detected by this work, their elite alleles generally conferred significantly higher averaged TGW values compared to their inferior counterparts. Statistical analysis of the comparisons was performed with SPSS for Windows 13.0.

## Results

### General Performance of the 94 Varieties

Natural rainfall differed markedly between the two experimental sites (HS and JY) during the 08/09 and 09/10 wheat crop cycles (Table S2). In general, the rainfall level in JY was substantially higher than that in HS during both cycles. No significant cold or frost damage was observed during the winter and early spring following sowing in autumn. Under the conditions with the IF treatment, all varieties were headed and harvested at times similar to the bulk wheat crop at the experimental locations (Table S2). Wheat growth was negatively affected under the RF, RN, and RP conditions, as exemplified by the decreases in plant height and effective number of tillers per square metre (Table S2). However, the heading and harvesting dates did not deviate substantially among the four conditions (Table S2), and sufficient seeds were harvested from all varieties under all four conditions.

### Assessment of TGW Data

The four cultivation treatments differing in water or fertiliser (nitrogen and phosphorus) supply were applied in each of three crop cycles (twice in HS and once in JY). This created a total of 12 different growth environments. As shown in Table 1, in each of the 12 environments, a broad variation in TGW was observed among the 94 varieties. The mean TGW values in JY were generally and significantly lower than those in HS under the IF, RN, and RP conditions, although such a difference was not found under the RF treatment. The treatment mean of TGW (TMTGW) was highest in the IF environment, and decreased significantly in the RF, RP, and RN environments, suggesting that the cultivation treatments were effective. Significant genotypic variance and relatively high broad sense heritability of TGW were found under all four treatments (Table 1).

**Table 1:** Comparison of thousand-grain weight (TGW) (g) across the 12 environments and an evaluation of treatment means (TMTGW), genotypic variance components (*δ* 2_G_) and broad sense heritabilities (*H* 2) of TGW in the 94 varieties cultivated under four different conditions.

Environment^1^	Min	Max	Mean^2^	TMTGW^3^	*δ* 2_G_	*H* 2
08/09HS-IF	35.7	55.7	45.7±4.1	45.6±4.4 a	13.8	0.81
09/10HS-IF	34.7	60.5	47.7±4.4			
09/10JY-IF	31.9	54.9	43.3±4.7*			
08/09HS-RF	27.2	46.5	35.5±3.5	37.1±3.8 c	11.2	0.74
09/10HS-RF	29.9	46.7	37.1±3.4			
09/10JY-RF	26.9	47.9	38.7±4.4			
08/09HS-RN	33.2	48.9	39.7±3.5	39.4±3.7 b	12.1	0.75
09/10HS-RN	30.3	50.2	40.3±3.7			
09/10JY-RN	28.3	46.9	38.2±3.9*			
08/09HS-RP	31.5	47.6	39.3±3.3	38.5±3.7 b	11.7	0.72
09/10HS-RP	28.5	50.6	39.6±3.9			
09/10JY-RP	25.7	46.4	36.6±4.4*			

1 The 12 environments were created by testing each of the four treatments (IF, RF, RN, and RP) in three crop cycles in Hengshui (HS) and Jiyuan (JY). IF, RF, RN, and RP refer to irrigated and fertilised, rainfed, reduced nitrogen, and reduced phosphorus treatments, respectively;

2 The mean TGW in JY was significantly lower than that in HS under the IF, RN, or RP conditions (*P*≤0.05);

3 Values marked with dissimilar letters were statistically significant (*P*≤0.05).

### Analysis of Molecular Markers and Construction of Genetic Linkage Map

A total of 426 SSR markers gave positive amplifications in the 94 varieties. Among these markers, 421 detected a single locus, and 5 detected multiple loci (≥2), resulting in 433 loci. In total, 3,330 alleles (ranging from 2 to 23 per locus) were detected. The average number of alleles per locus was 7.69. The PIC values of the markers varied from 0.02 to 0.89, with a mean of 0.516. The DNA samples of the 94 varieties were assayed by DArT, leading to the identification of 2,643 polymorphic DArT markers representing 2,643 loci. In all, 1823 DArT markers with *P*-values >80% were selected for GWA mapping. The PIC values estimated for the 1,823 DArTs ranged from 0.02 to 0.47, and the mean was 0.38. Among the 1,823 DArTs, 738 were located on the genetic map (Table S3), whereas the remaining 1,085 were not assigned.

A composite genetic linkage map was constructed carrying 1,171 loci (including 433 SSR and 738 DArT markers) (Table S3). The B genome contained the most loci (n = 587), followed by A (n = 355) and D genomes (n = 229). Group 3 chromosomes carried the largest number of loci (n = 261), followed by group 1 (n = 173), group 2 (n = 170), group 5 (n = 159), group 6 (n = 155), group 7 (n = 138), and group 4 (n = 115) chromosomes. The total genetic distance covered was approximately 3,762.9 cM, with a mean genetic distance of 3.92 cM between adjacent loci.

### Detection of Population Structure

**Figure 1:**
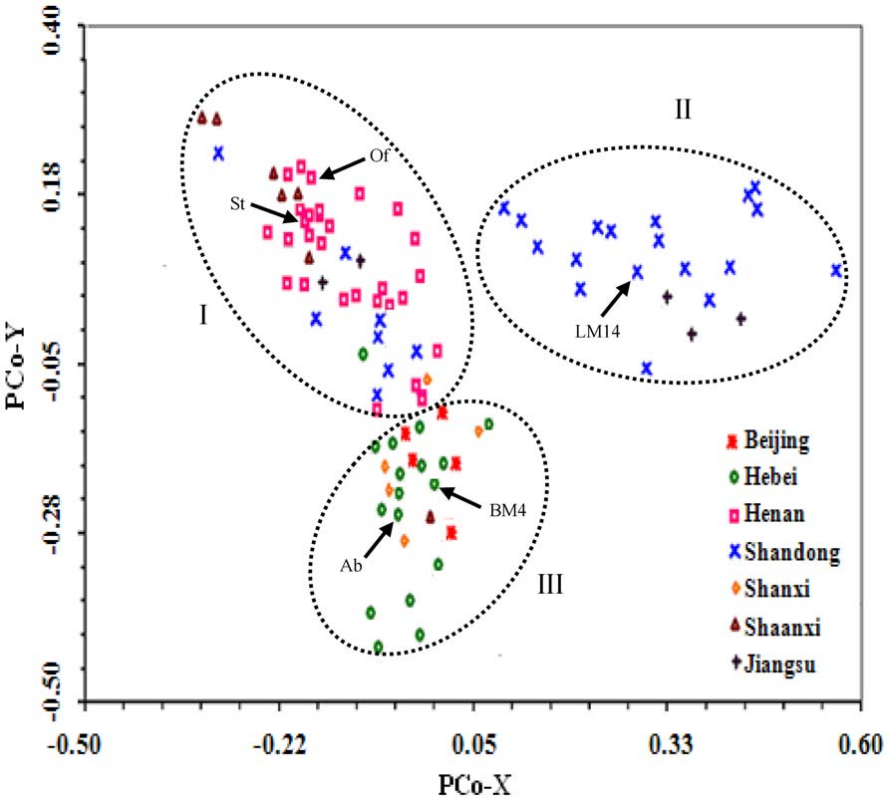
Distribution of the 94 elite common wheat varieties in two-dimensional PCoA space based on the genotyping data of 1,129 molecular markers. Three groups, I, II and III, were formed, and were dominated by the varieties from Henan, Shandong, and Hebei provinces, respectively. The core breeding parents in the three groups included Abbondanza (Ab), Bima 4 (BM4), Lumai 14 (LM14), Orofen (Of), and St (St2422–464).

The potential existence of subpopulations among the 94 varieties was detected using both PCoA analysis and the STRUCTURE program. In the former method, the 94 varieties were segregated into three groups differing mainly in geographic origin (Figure 1). Group I was dominated by the varieties from Henan province, group II by those from Shandong province, and group III by those from Hebei province. The five varieties from Beijing and four of the five varieties from Shanxi aggregated with those from Hebei (Figure 1), correlating with the close geographic relationships among Beijing, Shanxi, and Hebei [52]. Most of the varieties from Shaanxi clustered with those from Henan, and the varieties from Jiangsu were scattered in the Henan and Shandong groups (Figure 1). In each of the three subpopulations, there existed one or more founder lines frequently used as breeding parents (Figure 1). In the STRUCTURE analysis, the Δ*K* value was plotted against the number of hypothetical subgroups *K*, with the highest Δ*K* observed with *K = *3 (Figure S2). This indicated the existence of three subgroups in the genetic population used in this study, consistent with the PCoA analysis outcome.

### Investigation of Linkage Disequilibrium

**Figure 2:**
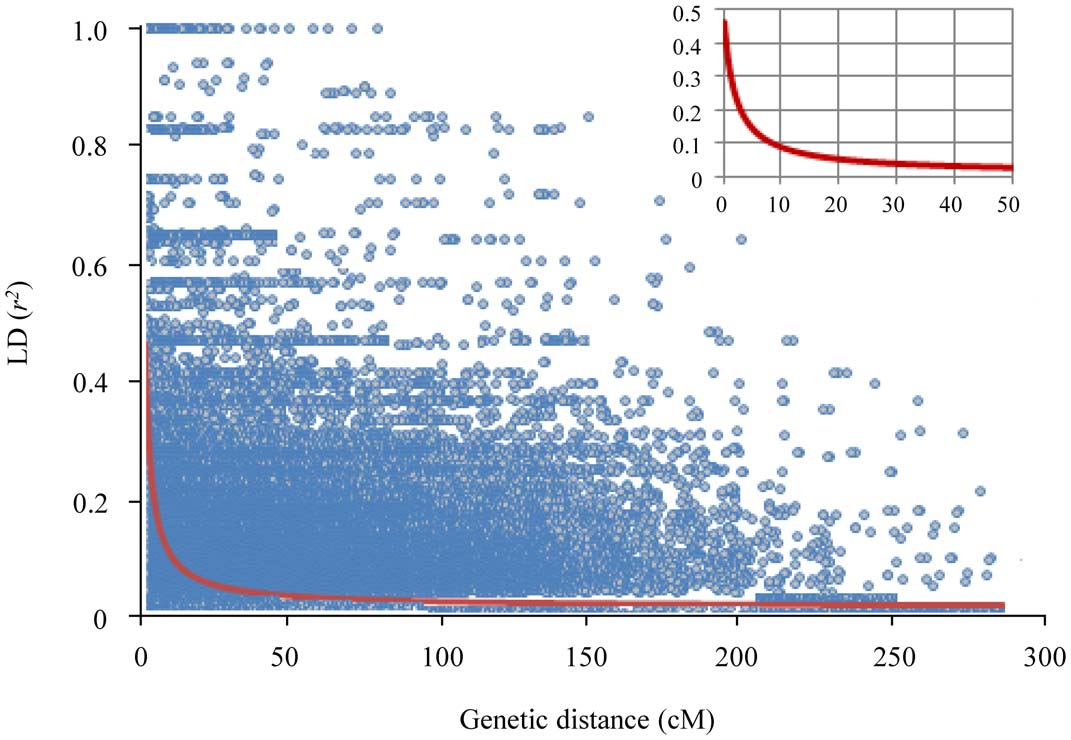
Linkage disequilibrium (LD, *r* ^2^) decay plot of 1,129 marker pairs as a function of genetic distance (cM) for the 94 common wheat lines used in this study. The inset provides a more detailed view of the LD decay characteristics over a 50 cM genetic distance.

The extent of LD was investigated using 1,129 loci (including 344, 574 and 211 loci from the A, B and D genomes, respectively). Pairwise LD, estimated using the squared allele frequency correlations (*r^2^*) at *P*<0.001, decayed rapidly with genetic distance (Figure 2). Approximately 45% of these comparisons had a significant LD (*P*<0.001), and the mean *r^2^* was 0.13 ranging from 0.034 to 1. The average LD decay distance was about 20 cM for locus pairs with *r^2^*>0.05 at the whole genome level. Some differences were observed in the extent of LD among the A, B and D genomes. For the B and D genomes, the LD decay distance was 15–20 cM for locus pairs with *r^2^*>0.05, but the corresponding value for the A genome was 20–25 cM. At the chromosomal level, chromosomes 2D, 4D, and 5D had 5–10 cM LD decay distances with *r^2^*>0.05. Seven chromosomes (1B, 2B, 3D, 5A, 5B, 7A, and 7D) showed higher LD decay distances (25–30 cM). The remaining 11 chromosomes had moderate LD decay distances of 10–20 cM.

### Chromosomal Loci Significantly Associated with TGW

Before embarking on a systematic GWA analysis, we evaluated the number of significantly associated loci (*P*<0.01) obtained using the different association models (GLM, GLM-Q, MLM-K, and MLM-Q-K) with the TGW data collected from the three environments with the IF treatment. This was carried out in order to assess the effectiveness of controlling population structure and/or kinship in reducing potential false positives. Among the four models, the number of positively associated loci was highest by the GLM (72–114), lowest by MLM-Q-K (15–20), and intermediate by GLM-Q (42–52) or MLM-K (39–56), demonstrating that both structure and relatedness were important for decreasing the probability of false positives in this study. Therefore, the GWA findings described below were obtained using MLM-Q-K.

Thirty-seven significantly associated signals, represented by 18 microsatellite and 19 DArT markers, were detected at *P*<0.01, with the percentage of variance explained by the associated markers (*R^2^*) ranging from 10.11% to 29.72% (Table S4). Most of these loci (n = 32) had known information on the chromosomal location and genetic linkage position, except for the five DArT loci listed in the lower section of Table S4. Five loci (*wPt-6965*, *Xgwm299*, *rPt-1806*, *Xpsp3152*, and *Xwmc17*) were associated with TGW across all 12 environments and were thus designated as environment-independent (EI) loci to facilitate further comparisons (Table 2). Eleven loci showed associations with TGW only in a particular set of environments with specific cultivation treatment and were tentatively referred to as environment-specific loci. Among the 11 loci, four (*Xgwm637*, *Xpsp3071, Xbarc1*, and *Xbarc235*) were stably associated with TGW only in the three environments with the IF treatment, whereas two *(Xwmc357* and *wPt-3426*), three (*Xgwm639*, *Xwmc486*, and *wPt-743515*) and two (*Xgwm666* and *Xcfd52*) were associated with TGW in the RF, RN, and RP treatment environments, respectively (Table 2). Additionally, two loci (*wPt-5432* and *wPt-2464*) were each stably associated with TGW in the eight HS environments irrespective of cultivation treatment, and similarly, *wPt-742096* and *wPt-742255* were each associated with TGW in the four JY environments (Table 2). These four loci affected TGW in a site-dependent manner. Compared to the 20 loci listed above, the other 17 loci exhibited less regular associations with TGW (Table S4). To concentrate on the major findings, we analysed in more detail the 20 loci regularly associated with TGW in all or specific sets of the 12 environments. Considering together the associations in the environment-independent or -specific manners, the total number of loci linked with TGW under IF, RF, RN and RP conditions were nine, seven, eight, and seven, respectively (Table 2).

**Table 2:** Phenotypic effects of the 20 chromosomal loci significantly associated with thousand-grain weight (TGW) in EI, ED or SD manners.

Locus and chromosomal		Elite allele^2^	AT^4^	Phenotypic effect (g)												Averaged effect (g)
location				09HS-IF	10HS-IF	10JY-IF	09HS-RF	10HS-RF	10JY-RF	09HS-RN	10HS-RN	10JY-RN	09HS-RP	10HS-RP	10JY-RP	
*Xgwm299*	3BL (87.78)^1^	205 bp (46)^3^	EI	1.9	2.41	1.41	2.06	1.14	1.54	2.39	2.24	2	2.47	3.37	1.37	2.03
*Xpsp3152*	6AL (80.66)	229 bp (13)		1.9	2.19	1.36	2.06	2.33	1.69	2.81	2.19	2.09	3	2.94	1.63	2.18
*Xwmc17*	7AL (89.20)	182 bp (17)		1.46	1.5	1.56	1.51	1.23	1.71	1.63	1.71	2.27	1.54	2.6	1.77	1.71
*wPt-6965*	3BS (10.28)	1 (22)		3.15	4.55	2.2	3.17	3.61	2.63	2.98	3.65	2.97	2.45	4.35	2.03	3.15
*rPt-1806*	3D	1 (22)		3.12	4.12	2.08	2.95	3.54	2.57	2.88	3.63	1.67	2.37	4	1.7	2.89
*Xgwm637*	4AL (93.86)	158 bp (10)	ED (IF)	1.56	2.39	1.39										1.78
*Xbarc1*	5AS (33.04)	276 bp (55)		1.39	1.71	1.29										1.46
*Xpsp3071*	6AL (95.09)	153 bp (9)		1.34	1.5	1.07										1.3
*Xbarc235*	7DL (160.57)	304 bp (55)		1.71	1.19	1.51										1.47
*Xwmc357*	5DL (80.63)	204 bp (42)	ED (RF)				1.96	1.94	1.47							1.79
*wPt-3426*	Unknown	1 (21)					2.55	3.31	1.93							2.6
*Xgwm639*	5DL (43.67)	168 bp (47)	ED (RN)							2.79	2.54	1.67				2.33
*Xwmc486*	6BS (6.05)	203 bp (17)								2.47	2.49	1.71				2.22
*wPt-743515*	Unknown	1 (40)								3.32	2.23	2.17				2.54
*Xgwm666*	5AL (73.41)	108 bp (34)	ED (RP)										2.1	1.77	1.79	1.89
*Xcfd52*	5DL (64.79)	281 bp (47)											2.29	1.49	1.33	1.7
*wPt-5432*	3BS (36.90)	1 (8)	SD (HS)	3.38	2		3.92	2.54		3.67	1.62		3.95	1.67		2.84
*wPt-2464*	3DS (14.41)	1 (25)		3.22	3.35		3.28	3.37		3.63	1.53		3.17	3.03		3.07
*wPt-742096*	Unknown	1 (71)	SD (JY)			1.98			1.96			2.5			2.65	2.27
*wPt-742255*	Unknown	1 (23)				1.88			1.92			2.52			2.2	2.13

1 The value in brackets indicates genetic distance (cM) along the given chromosome;

2 For the microsatellite marker, the size (bp) of the elite allele is given. For the diversity arrays technology (DArT) marker, the elite allele 1 indicates the presence of the locus;

3 The value in brackets indicates the number of varieties carrying the given elite allele in the association mapping population;

4 AT, association type; EI, ED and SD refer to environment-independent, environment-dependent, and site-dependent, respectively; IF, RF, RN, and RP indicate irrigated and fertilised, rainfed, reduced nitrogen, and reduced phosphorus treatments, respectively; HS, Hengshui; JY, Jiyuan.

### Phenotypic Effects of Associated Loci on TGW

**Figure 3:**
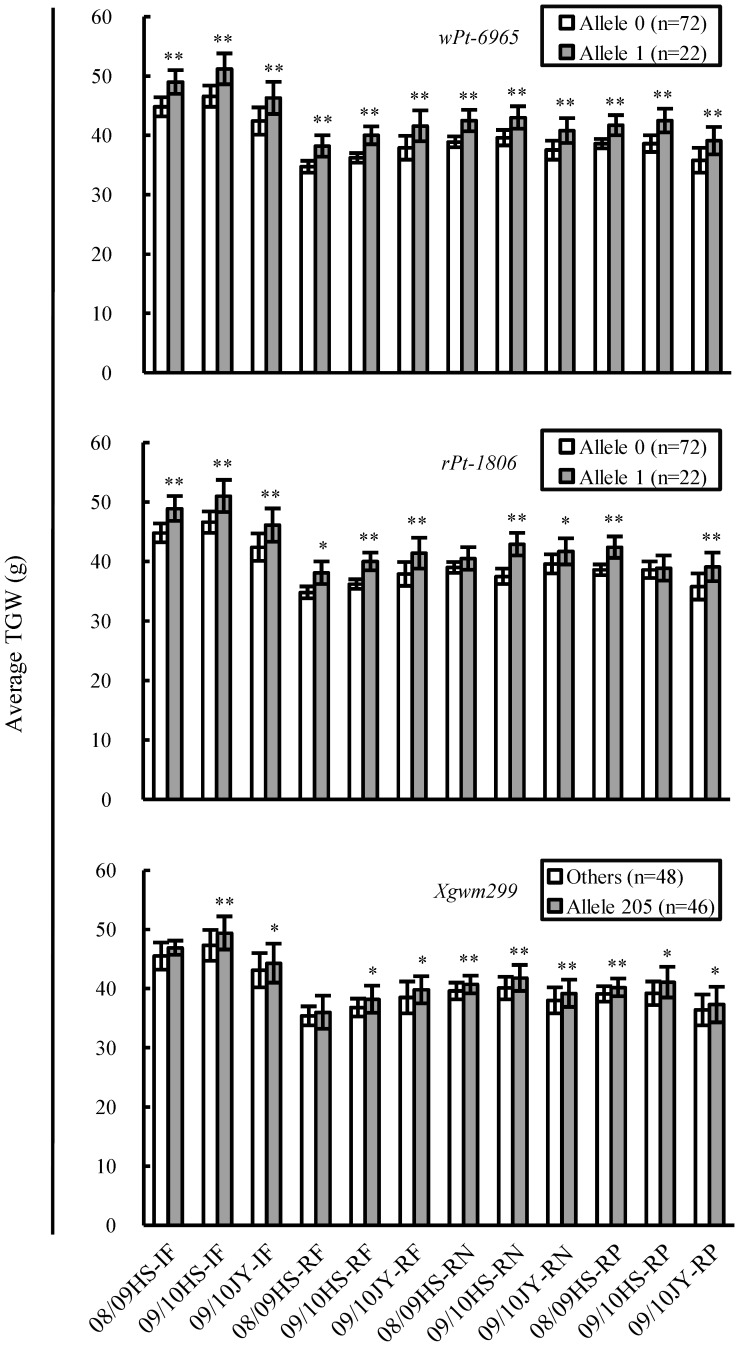
Phenotypic effects of the loci associated with thousand-grain weight (TGW) in an environment-independent (EI) manner analysed through marker allele-assisted genotyping. For the diversity arrays technology (DArT) loci (*wPt-6965* and *rPt-1806*), the elite alleles (indicated by Allele 1) refer to the presence of their respective DArT sequences. For the microsatellite locus (*Xgwm299*), the elite allele is represented by the actual size of specific amplicon (Allele 205). The inferior alleles of the DArT and microsatellite loci are indicated by “Allele 0” (reflecting the absence of the DArT sequence) and “others” (additional alleles with amplicon lengths differing from those of the elite alleles), respectively. Compared to the inferior alleles, the elite alleles of *wPt-6965*, *rPt-1806* and *Xgwm299* had generally positive effects on the average TGW (g) across all 12 environments.

Among the five EI loci, *wPt-6965* had the largest average phenotypic effect on TGW (3.15 g), followed by *rPt-1806* (2.89 g), *Xpsp3152* (2.18 g), *Xgwm299* (2.03 g) and *Xwmc17* (1.71 g) (Table 2). Through marker allele-assisted genotyping, it was found that the elite alleles of *wPt-6965*, *rPt-1806*, and *Xgwm299* conferred generally positive effects on TGW across all 12 environments, with the elite allele of *wPt-6965* being the most effective (Figure 3).

**Figure 4:**
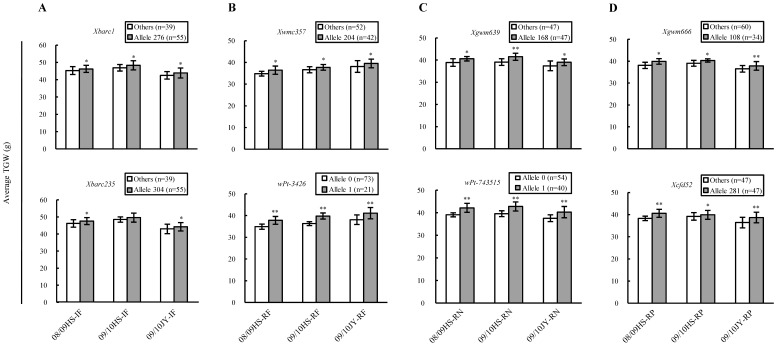
Phenotypic effects of the loci associated with thousand-grain weight (TGW) (g) in the irrigated and fertilised (IF), rainfed (RF), reduced nitrogen (RN), or reduced phosphorus (RP) environments as assessed through marker allele-assisted genotyping. For the microsatellite loci (*Xbarc1*, *Xbarc235*, *Xwmc357*, *Xgwm639*, *Xgwm666*, and *Xcfd52*), the elite alleles are represented by the actual size of specific amplicons (Allele 276, Allele 304, Allele 204, Allele 168, Allele 108, and Allele 281 for the six loci, respectively), with “Others” denoting inferior alleles. For the diversity arrays technology (DArT) loci (*wPt-3426* and *wPt-743515*), the elite (indicated by Allele 1) and inferior (represented by Allele 0) alleles refer to the presence and absence of their corresponding DArT sequences, respectively. (A-D) Comparisons of the phenotypic effects between the elite and inferior alleles of the associated loci on the average TGW across the three IF, RF, RN, or RP environments. The number of lines (n) in each varietal group is provided in brackets. *and **indicate statistical significance at *P*≤0.05 and 0.01, respectively.

Nine loci were associated with TGW in the three IF environments, including five EI and four IF-specific loci (*Xgwm637*, *Xpsp3071, Xbarc1*, and *Xbarc235*) (Table 2). The average phenotypic effects of the four IF loci (1.30–1.78 g) were generally lower than those of the five EI loci (1.51–3.30 g) under the IF treatment. Moreover, only the elite alleles of *Xbarc1* and *Xbarc235* were each found in more than 20 varieties (Table 2). Genotyping with *Xbarc1* or *Xbarc235* verified the beneficial effects of their elite alleles on the TGW, although such effects were not always significant across the three IF environments (Figure 4A). Interestingly, when the average TGWs recorded in the IF environments were compared to the corresponding values obtained under the RF, RN or RP conditions, the percentages of increase in the TGW were generally higher for the varietal groups carrying the elite alleles of *Xbarc1* or *Xbarc235* relative to those of the varietal groups with the inferior alleles of the two loci (Figure S3).

Five EI and two RF-specific (*Xwmc357* and *wPt-3426*) loci were detected to associate with TGW in the three RF environments (Table 2). The average phenotypic effects of *Xwmc357* (1.79 g) and *wPt-3426* (2.60 g) were lower than those of *wPt-6965* (3.02 g) and *rPt-1806* (3.14 g), approximately similar to those of *Xpsp3152* (2.03 g), and higher than those of *Xgwm299* (1.58 g) or *Xwmc17* (1.48 g), under the RF treatment (Table 2). Genotyping with *Xwmc357* or *wPt-3426* confirmed the positive effects of their elite alleles on TGW across the three RF environments, with that conferred by the *wPt-3426* elite allele being much larger (Figure 4B). When the average TGW values from the RF and IF conditions were compared, the varieties carrying the elite alleles of *Xwmc357* or *wPt-3426* displayed significantly lower decreases in the TGW than those with the inferior alleles of the two loci (Figure S4, left panel).

Five EI and three RN-specific (*Xgwm639*, *Xwmc486*, and *wPt-743515*) loci were found associated with TGW in the three RN environments (Table 2). The average phenotypic effects of *Xgwm639*, *Xwmc486*, and *wPt-743515* (2.22–2.54 g) were much smaller than that of *wPt-6965* (3.20 g), but close to those of the remaining four EI loci (1.87–2.73), under the RN treatment (Table 2). Genotyping with *Xgwm639* or *wPt-743515* verified the positive effects of their elite alleles on the TGW, with that conferred by the *wPt-743515* elite allele being slightly larger, across the three RN environments (Figure 4C). When the average TGWs from the RN and IF conditions were compared, the varieties carrying the elite alleles of *Xgwm639* or *wPt-743515* generally showed substantially less decreases in the TGW than those with the inferior alleles of the two loci (Figure S4, middle panel).

Among the five EI and two RP-specific (*Xgwm666* and *Xcfd52*) loci found associated with TGW in the three RP environments (Table 2), the average phenotypic effects of *Xgwm666* and *Xcfd52* (1.70–1.89 g) were comparable to that of *Xwmc17* (1.97 g), but considerably lower than those of the other four EI loci (2.40–2.94 g) (Table 2). Genotyping with *Xgwm666* or *Xcfd52* confirmed the positive effects of their elite alleles on TGW across the three RP environments (Figure 4D). Similarly, when the average TGWs from the RP and IF conditions were compared, the varieties carrying the elite alleles of *Xgwm666* or *Xcfd52* exhibited considerably less decreases in the TGW than did the varieties with the inferior alleles of the two loci (Figure S4, right panel).

**Figure 5:**
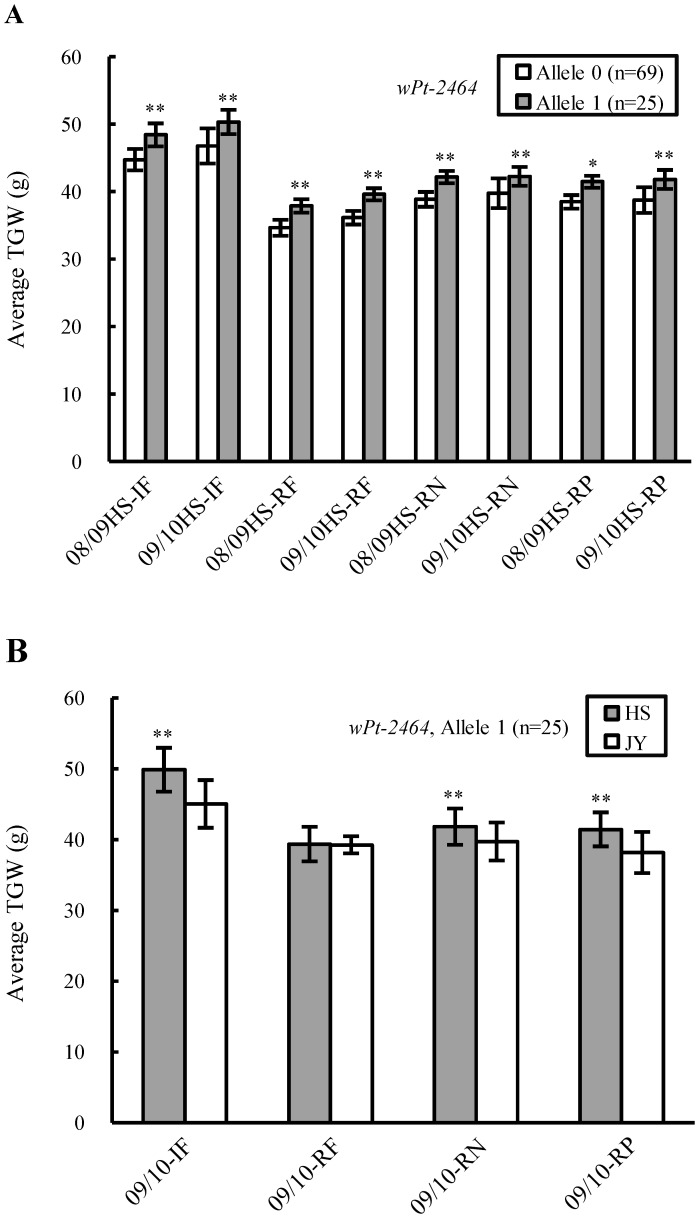
Phenotypic effects of *wPt-2464* associated with thousand-grain weight (TGW) (g) at the Hengshui (HS) experimental site analysed through marker allele-assisted genotyping. The elite and inferior alleles of *wPt-2464* are represented by “Allele 1” and “Allele 0”, respectively. *and **indicate statistical significance at *P*≤0.05 and 0.01, respectively. (A) Relative to the inferior allele, the elite allele conferred generally positive effects on the average TGW across the eight HS environments irrespective of cultivation treatment. The number of lines (n) in each varietal group is provided in brackets. (B) Comparisons of the average TGW values of the 25 varieties (carrying *wPt-2464* elite allele) cultivated in HS or Jiyuan (JY). The average TGWs of these varieties in HS were significantly higher than their corresponding values in JY in the irrigated and fertilised (IF), reduced nitrogen (RN), and reduced phosphorus (RP) environments. But such a difference was not observed in the rainfed (RF) environment.

The average phenotypic effects of the two loci (*wPt-5432* and *wPt-2464*) associated with TGW specifically in the eight HS environments were 2.84 g and 3.07 g, respectively (Table 2). Only the elite allele of *wPt-2464* was detected in more than 20 varieties (Table 2), and the positive effect of the elite allele of *wPt-2464* on TGW was confirmed by genotyping (Figure 5A). Furthermore, the average TGWs for the 25 varieties with the elite allele of *wPt-2464* were generally higher in HS than in JY under the IF, RN, and RP conditions, although such a difference in TGW was not found under the RF treatment (Figure 5B). In contrast, the average TGW did not vary substantially between HS and JY under the IF, RF, RN, and RP conditions for the 69 varieties with the inferior allele of *wPt-2464* (data not shown). The average phenotypic effects of the two loci (*wPt-742096* and *wPt-742255*) associated with TGW in the four JY environments were 2.27 g and 2.13 g, respectively (Table 2). Genotyping with *wPt-742096* or *wPt-742255* confirmed the beneficial effects of their elite alleles on the TGW across the four JY environments (Figure S5).

**Figure 6:**
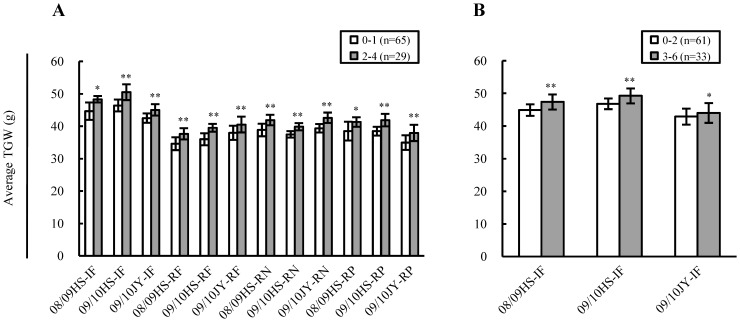
Assessing potential additive effects among the elite alleles of the associated loci on thousand-grain weight (TGW) (g). Assessment was carried out for the five loci associated with TGW in environment-independent (EI) manner and for the total number of loci associated with TGW in the irrigated and fertilised (IF), rainfed (RF), reduced nitrogen (RN), or reduced phosphorus (RP) environments. In each assessment, the 94 varieties were split into two varietal groups differing appropriately in the number of elite alleles. The number of lines (n) in each varietal group is provided in brackets. *and **indicate statistical significance at *P*≤0.05 and 0.01, respectively. (A) Assessing additive effects among the elite alleles of five EI loci. The highest number of EI elite alleles detected in a single variety was four. Therefore, the 94 varieties were split into two groups, with 0–1 and 2–4 elite alleles of the EI loci, respectively. The average TGW of the varietal group with 2–4 EI elite alleles was generally and significantly higher than that of the group with 0–1 EI elite allele across all 12 environments. (B) Examining additive effects among the elite alleles of the loci associated with TGW in the IF environments. Although the total number of associated loci under IF treatment was nine (including five EI and four IF specific loci), the highest number of elite alleles found in a single variety was only six. Thus, the 94 varieties were divided into two categories, carrying 0–2 and 3–6 elite alleles of the associated loci, respectively. The average TGW of the varietal group with 3–6 elite alleles was generally higher than that of the group with 0–2 elite alleles across the three IF environments. Using the same approach, substantial additive effects were also detected among the elite alleles of the loci associated with TGW under RF, RN, or RP treatments.

In addition to the analysis described above, efforts were also taken to assess potential additive effects among the elite alleles of the associated loci on TGW. For assessing such effects among the elite alleles of five EI loci, the 94 varieties were split into two groups, one with the elite alleles of 2–4 EI loci and the other carrying no elite allele of any EI loci or the elite allele of only one EI locus. The average TGW of the varieties with 2–4 elite alleles was generally and significantly higher than that of the varieties with no or only one elite allele in all 12 environments (Figure 6A). For investigating the additive effects among the nine loci associated with TGW in the three IF environments, the 94 varieties were divided into two categories, carrying 0–2 and 3–6 elite alleles of the associated loci, respectively. The average TGW of the varieties with 3–6 elite alleles was much higher than that of the varieties with 0–2 elite alleles across the three IF environments (Figure 6B). Using the same approach, substantial additive effects could also be detected among the elite alleles of the loci associated with TGW in the RF, RN, or RP environments (data not shown).

### Influences of 14 Associated Loci on Grain Length and Width Parameters

Considering the above data, we asked whether the associated loci could affect grain length and/or width. The potential influences of 14 loci, whose elite alleles were each found in more than 20 varieties (Table 2), on grain length and width were investigated. From Table 3, it is evident that the elite alleles of two EI loci (*wPt-6965* and *rPt-1806*) conferred significant increases in both grain length and width under all four cultivation conditions. In contrast, the elite allele of the EI locus *Xgwm299* improved only grain width. The elite alleles of the two IF-specific loci *Xbarc1* and *Xbarc235* caused significant improvements in grain length and width, respectively (Table 3). The elite alleles of *Xwmc357* and *wPt-3426*, associated with TGW specifically under the RF treatment, increased both grain length and width (Table 3). The elite alleles of *Xgwm639* and *wPt-743515*, associated with TGW specifically under the RN treatment, also improved both grain length and width (Table 3). The elite alleles of *Xgwm666* and *Xcfd52*, associated with TGW specifically under the RP treatment, increased grain width and grain length, respectively (Table 3).

**Table 3:** Positive influence of the elite alleles of 11 significantly associated loci on grain length (GL, mm) and grain width (GW, mm) in the irrigated and fertilised (IF), rainfed (RF), reduced nitrogen (RN), and reduced phosphorus (RP) environments in the 08/09 wheat crop cycle in Hengshui (HS).

Locus	Allele type	IF (HS)		RF (HS)		RN (HS)		RP (HS)	
		GL	GW	GL	GW	GL	GW	GL	GW
*wPt-6965*	Allele 1 (n = 22)^1^	6.86±0.17**^2^	3.51±0.12*	6.47±0.23**	3.22±0.15**	6.51±0.24**	3.34±0.15*	6.53±0.25**	3.34±0.13**
	Allele 0 (n = 72)	6.62±0.16	3.40±0.10	6.30±0.26	3.13±0.14	6.28±0.27	3.25±0.12	6.29±0.26	3.23±0.14
*rPt-1806*	Allele 1 (n = 22)	6.84±0.23**	3.50±0.12*	6.45±0.25**	3.20±0.16*	6.49±0.26**	3.33±0.16*	6.52±0.27**	3.34±0.15**
	Allele 0 (n = 72)	6.64±0.19	3.41±0.14	6.30±0.26	3.13±0.14	6.28±0.25	3.24±0.13	6.29±0.26	3.23±0.14
*Xgwm299*	Allele 205 bp (n = 46)	6.70±0.20	3.52±0.11*	6.37±0.19	3.29±0.12**	6.33±0.19	3.36±0.08*	6.35±0.20	3.31±0.13*
	Others (n = 48)	6.67±0.28	3.40±0.16	6.33±0.28	3.14±0.16	6.33±0.30	3.21±0.14	6.32±0.29	3.20±0.15
*Xbarc1*	Allele 276 bp (n = 55)	6.74±0.21*	3.45±0.16						
	Others (n = 39)	6.61±0.20	3.40±0.13						
*Xbarc235*	Allele 304 bp (n = 55)	6.71±0.26	3.49±0.14*						
	Others (n = 39)	6.65±0.23	3.38±0.15						
*Xwmc357*	Allele 204 bp (n = 42)			6.38±0.22*	3.20±0.14*				
	Others (n = 52)			6.30±0.20	3.10±0.15				
*wPt-3426*	Allele 1 (n = 21)			6.45±0.26**	3.22±0.16*				
	Allele 0 (n = 73)			6.30±0.25	3.11±0.14				
*Xgwm639*	Allele 168 bp (n = 47)					6.37±0.27*	3.31±0.12**		
	Others (n = 47)					6.29±0.28	3.22±0.14		
*wPt-743515*	Allele 1 (n = 40)					6.49±0.28**	3.33±0.16*		
	Allele 0 (n = 54)					6.29±0.27	3.25±0.12		
*Xgwm666*	Allele 108 bp (n = 34)							6.33±0.29	3.36±0.14**
	Others (n = 60)							6.31±0.26	3.15±0.16
*Xcfd52*	Allele 281 bp (n = 47)							6.39±0.21*	3.26±0.16
	Others (n = 47)							6.30±0.26	3.25±0.14

1 For the diversity arrays technology (DArT) locus, allele 1 was elite. For the microsatellite locus, the allele labelled with the specific fragment size (bp) was elite; “n” denotes the number of varieties carrying the given allele;

2 Statistical comparison was made between the averaged measurements of the two allele types. The asterisk and double asterisks indicate *P*≤0.05 (significant) or 0.01 (highly significant); Statistically significant comparisons are underlined.

Among the three loci associated with TGW in a site-dependent manner, the elite allele of *wPt-2464* improved both grain length and width across all four HS environments (Table S5). The elite allele of *wPt-742096* increased grain width more significantly than grain length in the four JY environments in the 09/10 wheat crop cycle (Table S6). In the same set of environments, the *wPt-742255* elite allele improved both grain length and width (Table S6). The influences on grain length and/or width of the 11 loci listed in Table 3 were also investigated with seed samples collected from an alternative environment (09/10-HS), and highly similar results were obtained. The positive effects of the elite allele of *wPt-2464* on grain length and width were also found with grains harvested from the four HS environments in the 09/10 crop cycle.

### Comparisons with Previously Identified Loci Affecting Grain Weight or Related Traits

For investigating the correspondences between our data and those of previous studies, we concentrated on the 15 associated loci with known information on chromosomal location and genetic position (Table 2), which was essential for achieving more accurate comparisons. Moreover, we considered only the correspondences that were based on the sharing of common markers with similar genetic positions on the linkage map (Table S7), because this type of strong correspondences may aid the identification of major chromosomal loci conserved in multiple genetic backgrounds and functioning in broad geographic areas for TGW control in common wheat.

Among the four EI loci (Table S7), three (*Xgwm299*, *Xpsp3152*, and *Xwmc17*) exhibited correspondences to previously reported loci affecting yield and related traits. *Xgwm299* was found in a previously reported grain weight QTL *QTgw.ipk-3B.2* in a Canadian environment [79], the meta-QTL *MQTL29* for yield and related traits [80], and a grain weight QTL in durum wheat [81]. Similarly, *Xwmc17* was involved in *MQTL49* for yield and related traits [80], and associated with grain weight [8]. Interestingly, *Xpsp3152* was implicated in a meta-QTL region (*MQTL_8*, *Xpsp3029*-*Xgwm570*-*Xpsp3071*-*Xpsp3152*-*Xwmc553*-*Xwmc179*) for grain length in multiple common wheat populations studied under UK conditions [35]. Furthermore, based on a published 6A linkage map [35], the genetic position of *Xpsp3152* was about 13 cM away from *Xcfd80*. The latter has been shown closely linked with *TaGW2-6A*, which plays an important role in determining the grain width and weight of common wheat [7].

Among the four IF-specific loci (Table S7), *Xgwm637* was found in two previously reported yield QTL regions, namely *Xgwm637*-*Xgwm2228* (*MQTL32*) in common wheat [80] and *Xgwm637*-*wPt6515*-*wPt7558* in durum wheat [82]. *Xpsp3071* was involved in two QTL regions (*Xcwm306*-*Xpsp3071* and *Xpsp3071*-*Xgwm570*) for grain weight in Chinese wheat [30], *MQTL_8* controlling grain length in several wheat populations [35], and a QTL region (*Xwmc32*-*Xpsp3071*) for grain weight and yield in a UK environment [83]. Of the two RN-specific loci (Table S7), *Xgwm639* was reported in a QTL for straw nitrogen content [28], and two QTL regions for grain weight (*Xcfd8*-*Xgwm639*) or glutamine synthetase activity (*Xgwm639*-*Xcfd12*) in a French environment [29]. *Xwmc486* has been found associated with heading date and peduncle length in durum wheat [45]. Regarding the two RP-specific loci (*Xgwm666* and *Xcfd52*, Table S7), only *Xgwm666* exhibited relevant correspondences to previously identified QTLs. It was implicated in a meta-QTL (*MQTL_6*, *Xwmc492-Xgwm666*) for grain length and width [35], a meta-QTL (*MQTL41*, *Xwmc327-Xgwm666*) for yield related traits [80], and a QTL region (*Xgwm666-Xgwm271*) for shoot phosphorus uptake and utilisation efficiency [84].

Compared to the eight loci described above, the remaining seven loci (*wPt-6965*, *Xbarc1*, *Xbarc235*, *Xwmc357*, *Xcfd52*, *wPt-5432*, and *wPt-2464*, Table S7) did not display strong correspondences to previously studied loci affecting grain weight and related traits.

### Genetic Relationships Among *Xpsp3071*, *Xpsp3152*, and *TaGW2-6A*

**Figure 7:**
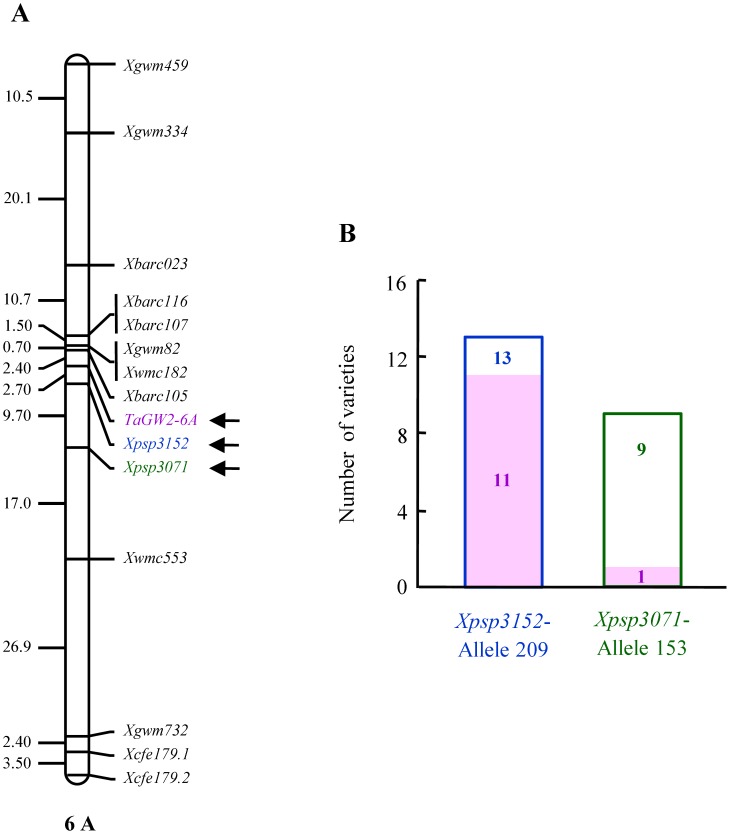
Investigation of the genetic relationships among *Xpsp3152*, *Xpsp3071*, and *TaGW2-6A*. (A) The relative genetic positions of *Xpsp3152*, *Xpsp3071*, and *TaGW2-6A* (indicated by arrows) on chromosome 6A mapped with the DH population of Huapei 3× Yumai 57 [70]. The genetic distance (cM) between adjacent markers is shown on the left side of the diagram. (B) Analysis of potential co-segregation between the larger grain haplotype (6A–A) of *TaGW2-6A* and the elite alleles of *Xpsp3152* or *Xpsp3071*. Among the 13 varieties carrying the elite allele of *Xpsp3152* (Allele 229), 11 had the 6A–A haplotype of *TaGW2-6A*. By contrast, among the nine varieties with the elite allele of *Xpsp3071* (Allele 153), only one had the 6A–A haplotype of *TaGW2-6A*.

In the linkage maps published for 6A chromosome [35], [85], [86], *Xpsp3071* and *Xpsp3152* are usually located close to each other, with the distance between them being less than 10 cM. The finding that both *Xpsp3071* and *Xpsp3152* are involved in the meta-QTL *MQTL_8* controlling grain length [35], the indirect evidence that *Xpsp3152* might reside proximal to *TaGW2-6A* (see above), and the associations of *Xpsp3071* and *Xpsp3152* with TGW in different manners (Table 2) prompted us to further investigate the genetic relationships among *Xpsp3071*, *Xpsp3152*, and *TaGW2-6A*. Using the DH population derived from two common wheat varieties Huapei 3 and Yumai 57 (both included in our association mapping population), we determined the relative genetic positions among *Xpsp3071*, *Xpsp3152*, and *TaGW2-6A* on 6A chromosome. As shown in Figure 7A, the genetic distance from *TaGW2-6A* to *Xpsp3152* (2.7 cM) was considerably shorter than that between *TaGW2-6A* and *Xpsp3071* (12.4 cM). After genotyping with a SNP marker developed based on *TaGW2-6A* nucleotide sequence polymorphism [7], it was found that 11 of the 13 varieties carrying the elite allele of *Xpsp3152* also had the 6A–A haplotype of *TaGW2-6A* (Figure 7B). This haplotype has previously been observed to associate with larger grain weight [7]. On the other hand, among the nine varieties with the elite allele (153 bp) of *Xpsp3071*, only one had the 6A-A haplotype of *TaGW2-6A* (Figure 7B), and the remaining eight carried the 6A–G haplotype of *TaGW2-6A* that is linked to smaller grain weight [7]. These data revealed that the elite allele of *Xpsp3152* segregated with the larger grain haplotype of *TaGW2-6A* substantially more frequently than the elite allele of *Xpsp3071* did in the current mapping population.

## Discussion

### Main Structural and LD Features of the Studied Elite Wheat Population

Structural analysis, conducted with two complementary methods, suggested that the elite wheat varieties used in this work fell into three subpopulations. This is consistent with the general observations on the existence of genetic differences among common wheat varieties or landraces [38]. Sharing of highly similar gene alleles for adapting to specific ecological regions and common parentage have been suggested as the main causes for subpopulation differentiation in cereal crops [38]. These causes are also likely important for the subpopulation differentiation observed in our mapping population because of the following reasons. First, the three subpopulations were composed mainly of the varieties from Henan, Hebei and Shandong provinces, respectively. These three regions differ from each other in both climate and soil conditions [87]. Second, for each subpopulation, there usually existed one or more founder parents that have been used extensively for wheat breeding in the province to which the subpopulation belong. Resolving the population structure character and its incorporation into the marker-trait association analysis improved precision for identifying the significantly associated loci in this study.

Investigations at the genome, subgenome and chromosomal levels suggested that the LD decay distance in the current elite wheat population was 5–30 cM. On the basis of this LD range and the average marker interval (3.92 cM) calculated from our composite genetic map, we can reasonably suggest that the GWA analysis described here is likely efficient for detecting genome-wide marker-trait associations. The genome-wide LD decay distance of our mapping population (20 cM on average) is larger than that estimated for a different panel of improved common wheat varieties (5–10 cM) [88]. The varieties used in the previous study were more diverse in the geographic origin (coming from all ten wheat cultivation zones in China), and the timing of release (from the 1940s to the 1990s) [88], [89]. By contrast, the 94 varieties in our mapping population were less diverse in geographic origin (largely from the Yellow and Huai river valley winter wheat zone), and timing of release (from the 1980s to the 2000s). The common wheat varieties developed during the 2000s generally have higher yields than those released prior to 2000 but with reduced genetic diversity [52]. Thus, the genetic diversity in our mapping population may be lower, possibly accounting for the higher LD observed. The same reason may also, at least partly, account for the higher LD decay distances recorded by us for the A, B, and D subgenomes (15–25 cM) and the 21 chromosomes (5–30 cM) when compared to the corresponding values in the previous study [88]. Nonetheless, similar to the data reported previously [88], we also found that the average LD decay distance of subgenome A was much higher than that of subgenomes B and D, and that 2B, 5A, 7A, and 7D appeared to have more elevated LD decay distances compared to other chromosomes.

### New Insights Regarding TGW Control in Common Wheat

Like previous association mapping studies in wheat (e.g., [8], [33], [46], [48]–[51]), a fairly large number of significant loci was identified by this work, suggesting that TGW is genetically controlled in a complex manner under the conditions examined here. However, the present work is unique in that we simultaneously revealed the chromosomal loci involved in TGW control in common wheat grown under IF, RF, RP, and RN conditions. The *R^2^* values (ranging from 10.11% to 29.72%, Table S4) modeled for the associated markers (loci) were relatively large, which is consistent with the high inheritability of TGW under the four conditions examined (Table 1). The total phenotypic variance explained by the associated markers (loci) exceeded 100% for each of the 12 environments. This is likely caused by the existence of significant LD between certain markers, which leads to overestimations of *R^2^*. Based on a combined consideration of the environmental specificities and the phenotypic effects of the 20 loci and their correspondences to previously described genetic determinants of grain weight and related traits in wheat, the following new insights may be summarised.

First, there exist multiple chromosomal loci that are stably associated with common wheat TGW under IF, RF, RP and RN conditions, which may contribute positively to the genetic control of TGW in diverse environments. Among five such loci characterised in this work (Table 2), *Xgwm299*, *Xpsp3152*, and *Xwmc17* may represent major genetic determinants important for grain weight in the common wheat varieties cultivated in many countries, because they showed strong correspondence to a number of previously identified QTLs involved in the control of grain weight and related traits under British, Canadian or Chinese conditions (Table S7). Interestingly, *Xgwm299* also corresponded directly to a grain weight QTL detected in durum wheat (Table S7), indicating that it may be involved in grain weight control in both durum and common wheats. Second, there are multiple loci that are associated with common wheat TGW in the IF, but not RF, RN, and RP, environments. Among four such loci investigated in this work (Table 2), *Xgwm637* corresponded directly to the previously discovered QTLs for yield and related traits in durum and common wheats (Table S7), indicating that it may be involved in grain weight and yield control in both types of wheat crops under IF conditions. Consistent with previous findings [30,35,83, Table S7], we observed that *Xpsp3071* was associated with TGW, suggesting that this locus is probably important for grain weight control in the IF environments in different countries. Third, there also exist a couple of chromosomal loci that are regularly associated with common wheat TGW in the RF, RN, or RP environments, but not under the conditions with the IF treatment (Table 2). Based on our data and those published previously (Table S7), *Xgwm639* and *Xgwm666* are likely to be broadly important for grain weight control in the environments with reduced applications of nitrogen or phosphorus fertilisers. Fourth, there are chromosomal loci (e.g., *wPt-2464* and *wPt-5432*) that are stably associated with common wheat TGW in a site-dependent manner, and their associations with TGW in the given location are not affected by the IF, RF, RN or RP treatments. The phenotypic effects of *wPt-2464* were investigated in more detail in this work, and found to contribute substantially to the TGW difference between the two experimental sites (Hengshui and Jiyuan) under the IF, RN, and RP conditions. Finally, because the total number of loci associated with TGW under the IF, RF, RN and RP conditions was nine, seven, eight, and seven, respectively (Table 2), and the mean TGW was highest for IF, intermediate for RP and RN, and lowest for RF environments (Table 1), we speculate that higher TGW may involve the function of more chromosomal loci related to the genetic control of this trait.

From the discussion above, it is clear that some of the loci revealed in this work may represent conserved chromosomal regions important for grain weight control in multiple genetic backgrounds and broad geographic areas. Because of a lack of strong correspondence to grain weight or yield QTLs reported in the past, *wPt-6965*, *Xbarc1*, *Xbarc235*, *Xwmc357*, *Xcfd52*, *wPt-5432*, and *wPt-2464* may represent newly identified chromosomal loci for TGW control in common wheat. However, further research is needed to verify this possibility. Although this study was conducted in 12 environments with four different cultivation treatments, it is likely that the major loci found in this work may also be associated with grain weight (or other traits) under alternative conditions that were not examined in this study. Thus, additional work is needed to evaluate the effects of these loci on grain weight in more diverse environments. One major TGW locus (*Xwmc17*) was found both by us and an earlier association mapping study of grain weight in Chinese common wheat [8]. However, many of the associated loci were different between the two studies, possibly owing to differences in the mapping population (see above), the number and type of molecular markers, and the test environments deployed for the association analysis. Nevertheless, our data, plus those reported earlier [8], represent a more complete understanding of the loci involved in the grain weight control of Chinese common wheat germplasm lines and elite varieties.

### New Information on the Mechanisms Involved in the Function of TGW Loci

As well as identifying significantly associated loci, this work also generated some new information on the mechanisms involved in the function of the loci in controlling common wheat grain weight. First, there appears to be a general tendency that the environmentally stable and specific loci may act additively to increase TGW under the IF, RF, RP, and RN conditions (Figure 6). This is consistent with the proposition that higher TGW in common wheat may involve the function of more chromosomal loci acting on the genetic control of grain weight (see above). Second, it is likely that most of the associated loci may affect common wheat TGW through influencing grain size, because the allelic variations of 14 loci were linked with differences in grain length, width or both (Table 3, Tables S5 and S6). Finally, there may be genetic, and possibly functional, linkages between the major grain weight loci *Xpsp3152* and *TaGW2-6A*, which were located closely to each other on the 6A linkage map. Our observation agrees with the previous findings on the participation of *GW2* in the control of grain width and weight in rice, maize, and common wheat [7], [90]–[92], and may facilitate further characterisation and application in molecular breeding of this important locus. In contrast, *Xpsp3071* may function differently from *Xpsp3152* and *TaGW2-6A*, because it was associated with TGW under more specific conditions (relative to *Xpsp3152*), and its elite allele did not segregate closely with that of *TaGW2-6A*.

### Potential Application of the Associated Loci for Improving Common Wheat Grain Weight Trait

The major TGW loci revealed in this study may be of practical value for further improving the wheat grain weight trait to efficiently utilise water, nitrogen, and phosphorus resources under optimal or unfavourable conditions. This is possible for the following reasons. First, for most of the 20 loci, their elite allele was generally found in less than 50% of the 94 varieties (Table 2). None of the 94 varieties carried elite alleles for the whole set of loci found regularly associated with TGW in the IF (n = 9), RF (n = 7), RN (n = 8), or RP (n = 7) environments. These data suggest a large potential for combining the elite alleles of the 20 loci in suitable varietal backgrounds to increase grain weight and yield potential. Second, as discussed above, additive effects exist among the TGW loci, the pyramiding of which will likely lead to further increases in grain weight. Third, the elite alleles of many of the TGW loci uncovered here had positive and complementary effects on grain length and width. Their combination may lead to an enlargement of seed size and thus grain weight. Finally, the elite allele of *Xwmc17*, which was found associated with grain weight here and previously [8], has already been widely used in the development of high-yielding common wheat varieties for the 10 wheat cultivation zones in China [8]. It has been demonstrated that the common wheat lines selected for higher grain weight and yield potential often have enriched QTLs for TGW and other yield components [26], confirming that TGW loci and their pyramiding are likely useful for increasing wheat grain weight and productivity.

In summary, we have identified, and analysed in more detail, 20 genomic loci that were associated with common wheat grain weight under all 12, or specific sets of, cultivation environments differing in water and fertiliser levels. These loci provide new knowledge on the genetic determinants and the mechanisms involved in grain weight control of common wheat. They also represent potentially new marker resources valuable for further improvements in grain weight and yield traits in common wheat. We are now in the process of verifying the effects of these loci on TGW with a wider collection of common wheat varieties and in more diverse environments. Efforts are also being made to pyramid these TGW loci in high- yielding and well-adapted varietal backgrounds through marker-assisted selection.

## Supporting Information

Figure S1 **A diagram showing the ten wheat cultivation zones in China, and locations of the two experimental sites, Hengshui (HS) and Jiyuan (JY), in zone II.** The designations of the ten zones are as follows. I, northern winter wheat zone; II, Yellow and Huai river valleys facultative wheat zone; III, middle and low Yangtze valleys autumn-sown spring wheat zone; IV, southwestern autumn-sown spring wheat zone; V, southern autumn-sown spring wheat zone; VI, northeastern spring wheat zone; VII, northern spring wheat zone; VIII, northwestern spring wheat zone; IX, Qinghai-Tibetan Plateau spring-winter wheat zone; X, Xinjiang winter-spring wheat zone. The diagram is modified from [8].
(TIF)

Figure S2 **Analysis of population structure using STRUCTURE software (v2.2).** The value of ΔK peaked at 3, indicating three subpopulations in the association mapping population.
(TIF)

Figure S3 **Analysis of the enhancement effects on thousand-grain weight (TGW) (g) by the elite alleles of** ***Xbarc1*** **and** ***Xbarc235*** **under well-resourced (irrigated and fertilised, IF) conditions through marker allele-assisted genotyping.** The elite alleles of *Xbarc1* and *Xbarc235* are 276 (Allele 276) and 304 (Allele 304) bp, respectively. “Others” refers to inferior alleles. The average TGWs from the IF environments were compared to the corresponding values obtained under the rainfed (RF), reduced nitrogen (RN), and reduced phosphorus (RP) conditions. The percentage increases in TGW were generally higher for the varietal groups carrying the elite alleles of *Xbarc1* or *Xbarc235* relative to those of the varietal groups with the non-elite alleles of the two loci. The number of lines (n) in each varietal group is provided in brackets. *and **indicate statistical significance at *P*≤0.05 and 0.01, respectively.
(TIF)

Figure S4 **Assessment of tolerance to the decrease in thousand-grain weight (TGW) (g) conferred by the elite alleles of several associated loci under the rainfed (RF) (left panel), reduced nitrogen (RN) (middle panel), or reduced phosphorus (RP) (right panel) conditions.** For the diversity arrays technology (DArT) loci (*wPt-3426* and *wPt-743515*), the elite alleles (indicated by Allele 1) refer to the presence of their corresponding DArT sequences. For the microsatellite loci (*Xwmc357*, *Xgwm639*, *Xgwm666*, and *Xcfd52*), the elite alleles are represented by the actual size of specific amplicons (Allele 204, Allele 168, Allele 108, and Allele 281 for the four loci, respectively). “Allele 0” and “Others” are inferior alleles. The average TGWs from the RF (RN or RP) environments were compared to the corresponding values obtained under the IF conditions. Varieties carrying the elite alleles generally exhibited much less decreases in TGW than those with the inferior alleles. The number of lines (n) in each varietal group is provided in brackets. *and **indicate statistical significance at *P*≤0.05 and 0.01, respectively.
(TIF)

Figure S5 **Evaluation of the phenotypic effects of** ***wPt-742096*** **and** ***wPt-742255*** **associated with thousand-grain weight (TGW) at the Jiyuan (JY) experimental site through marker allele-assisted genotyping.** The elite and inferior alleles of the two diversity arrays technology (DArT) loci are represented by “Allele 1” and “Allele 0", respectively. Relative to the inferior alleles, the elite alleles of *wPt-742096* and *wPt-742255* generally had positive effects on the average TGW (g) across the four JY environments irrespective of cultivation treatment. The number of lines (n) in each varietal group is provided in brackets. *and **indicate statistical significance at *P*≤0.05 and 0.01, respectively.
(TIF)

Table S1 **Schemes of water and fertiliser supply in the four cultivation treatments tested in this study.**
(DOC)

Table S2 **Differences in natural rainfall between Hengshui (HS) and Jiyuan (JY) and general performance of the 94 varieties under the irrigated and fertilised (IF), rainfed (RF), reduced nitrogen (RN), and reduced phosphorus (RP) conditions during the 08/09 and 09/10 wheat crop cycles.**
(DOC)

Table S3 **Composite linkage maps of 21 common wheat chromosomes.**
(XLS)

Table S4 **A list of 37 chromosomal loci significantly associated with thousand-grain weight (TGW) detected using the mixed linear model with controls for population structure and kinship (MLM-Q-K).**
(XLS)

Table S5 **Positive influence of** ***wPt-2464*** **elite allele on grain length (GL, mm) and grain width (GW, mm) in the irrigated and fertilised (IF), rainfed (RF), reduced nitrogen (RN), and reduced phosphorus (RP) environments during the 08/09 wheat crop cycle in Hengshui (HS).**
(DOC)

Table S6 **Positive influence of** ***wPt-742096*** **and** ***wPt-742255*** **elite alleles on grain length (GL, mm) and grain width (GW, mm) in the irrigated and fertilised (IF), rainfed (RF), reduced nitrogen (RN), and reduced phosphorus (RP) environments during the 09/10 wheat crop cycle in Jiyuan (JY).**
(DOC)

Table S7 **Comparisons of the 15 significantly associated loci found in this work with previously identified loci affecting grain, yield, and related traits.**
(DOC)
