# Supplementary figures and images for: Association Analysis of Genomic Loci Important for Grain Weight Control in Elite Common Wheat Varieties Cultivated with Variable Water and Fertiliser Supply
Source: PLoS One. 2013 Mar 4;8(3):e57853. doi: 10.1371/journal.pone.0057853 (PMC3587626; doi:10.1371/journal.pone.0057853)

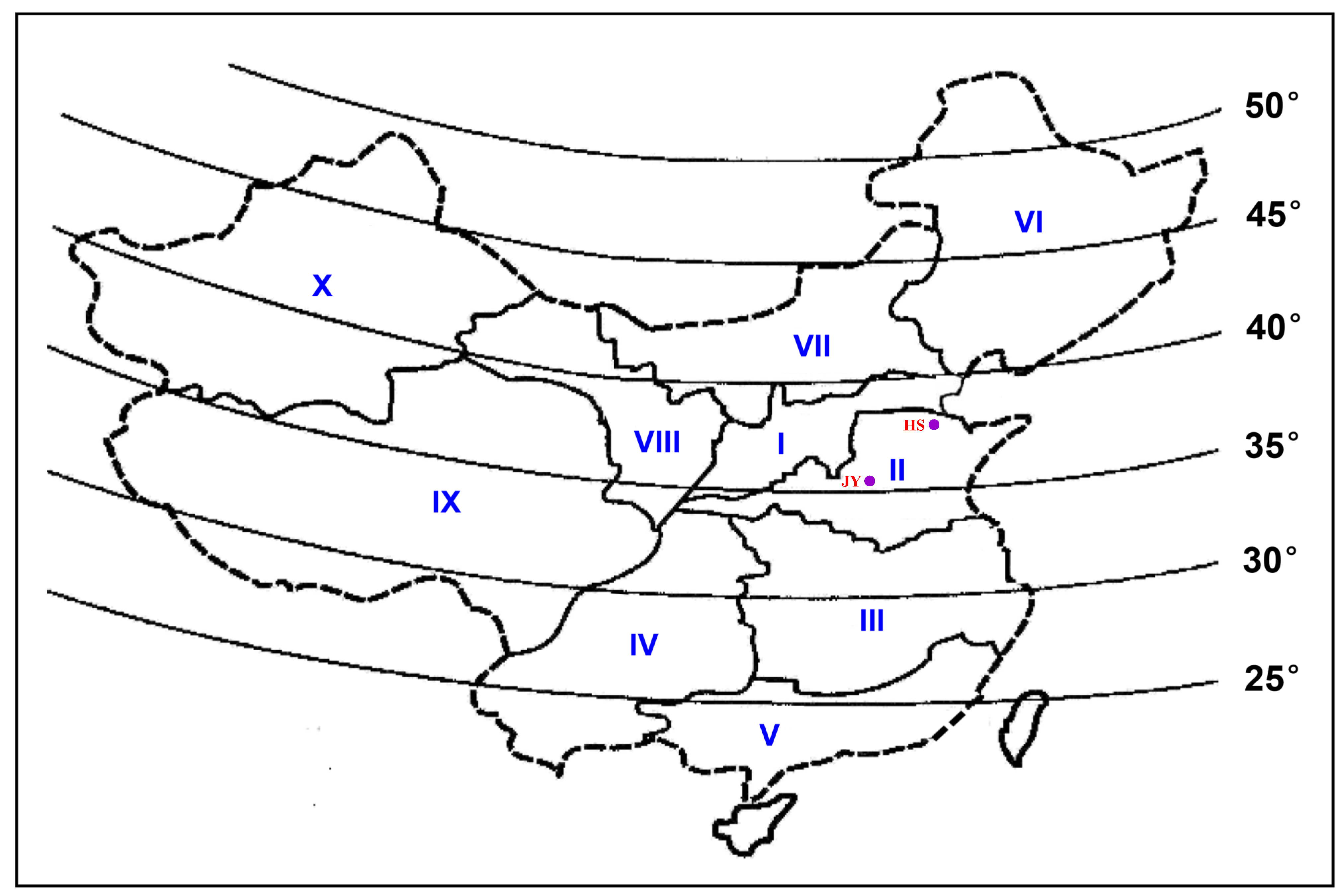

Supplement: Figure S1 — A diagram showing the ten wheat cultivation zones in China, and locations of the two experimental sites, Hengshui (HS) and Jiyuan (JY), in zone II. The designations of the ten zones are as follows. I, northern winter wheat zone; II, Yellow and Huai river valleys facultative wheat zone; III, middle and low Yangtze valleys autumn-sown spring wheat zone; IV, southwestern autumn-sown spring wheat zone; V, southern autumn-sown spring wheat zone; VI, northeastern spring wheat zone; VII, northern spring wheat zone; VIII, northwestern spring wheat zone; IX, Qinghai-Tibetan Plateau spring-winter wheat zone; X, Xinjiang winter-spring wheat zone. The diagram is modified from [8]. (TIF) [file pone.0057853.s001.tif]

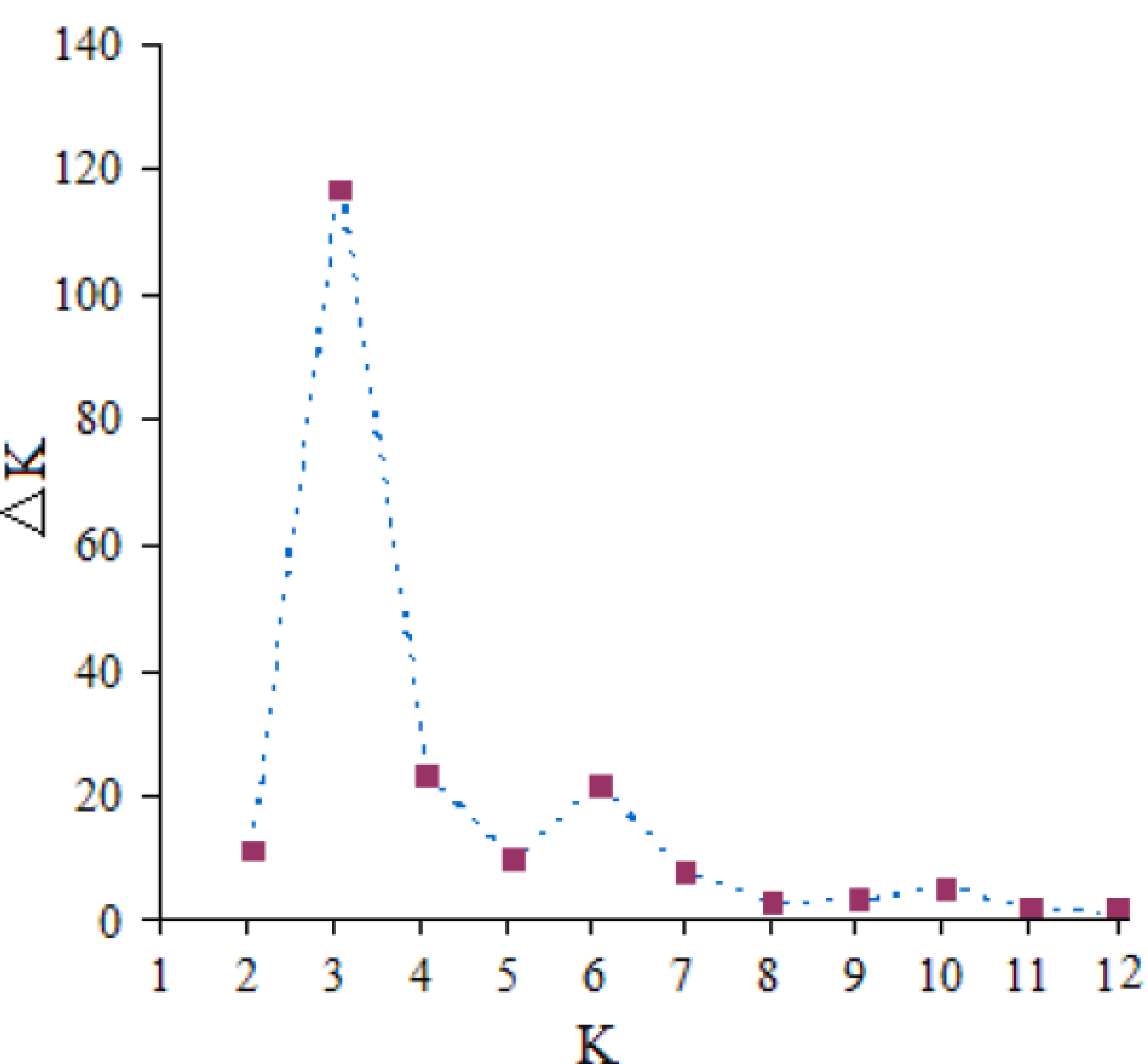

Supplement: Figure S2 — Analysis of population structure using STRUCTURE software (v2.2). The value of ΔK peaked at 3, indicating three subpopulations in the association mapping population. (TIF) [file pone.0057853.s002.tif]

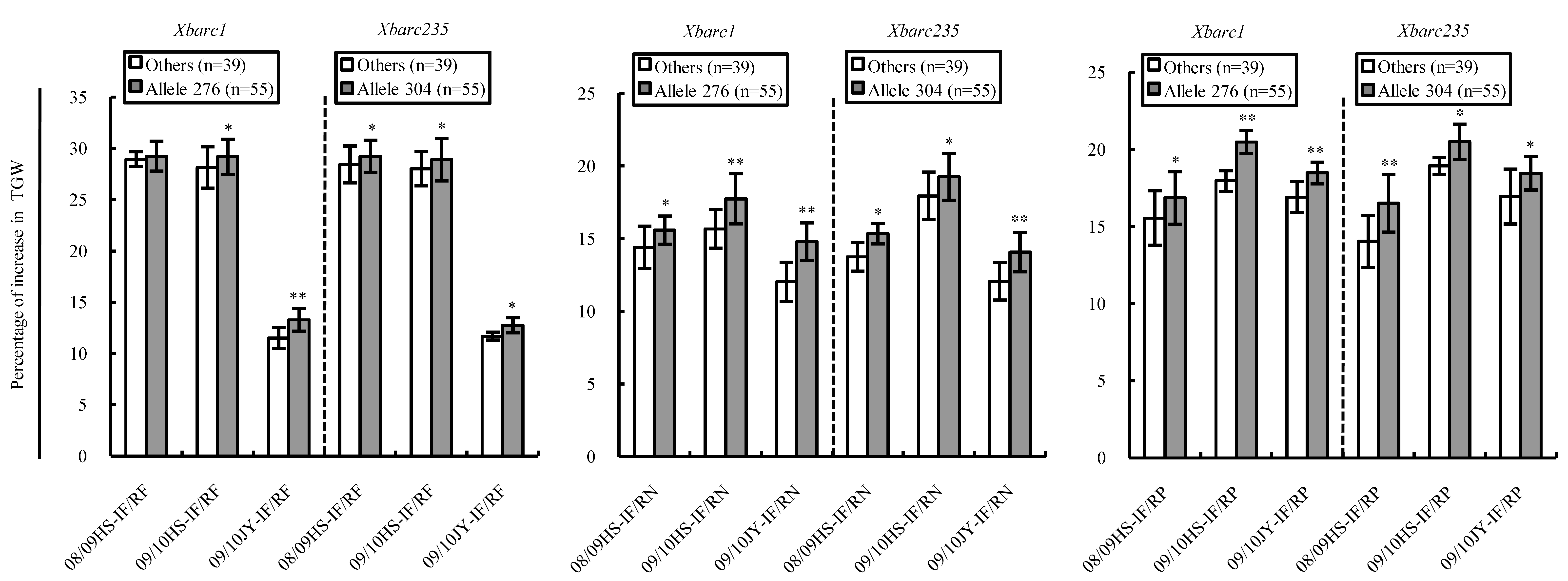

Supplement: Figure S3 — Analysis of the enhancement effects on thousand-grain weight (TGW) (g) by the elite alleles of Xbarc1 and Xbarc235 under well-resourced (irrigated and fertilised, IF) conditions through marker allele-assisted genotyping. The elite alleles of Xbarc1 and Xbarc235 are 276 (Allele 276) and 304 (Allele 304) bp, respectively. “Others” refers to inferior alleles. The average TGWs from the IF environments were compared to the corresponding values obtained under the rainfed (RF), reduced nitrogen (RN), and reduced phosphorus (RP) conditions. The percentage increases in TGW were generally higher for the varietal groups carrying the elite alleles of Xbarc1 or Xbarc235 relative to those of the varietal groups with the non-elite alleles of the two loci. The number of lines (n) in each varietal group is provided in brackets. *and **indicate statistical significance at P≤0.05 and 0.01, respectively. (TIF) [file pone.0057853.s003.tif]

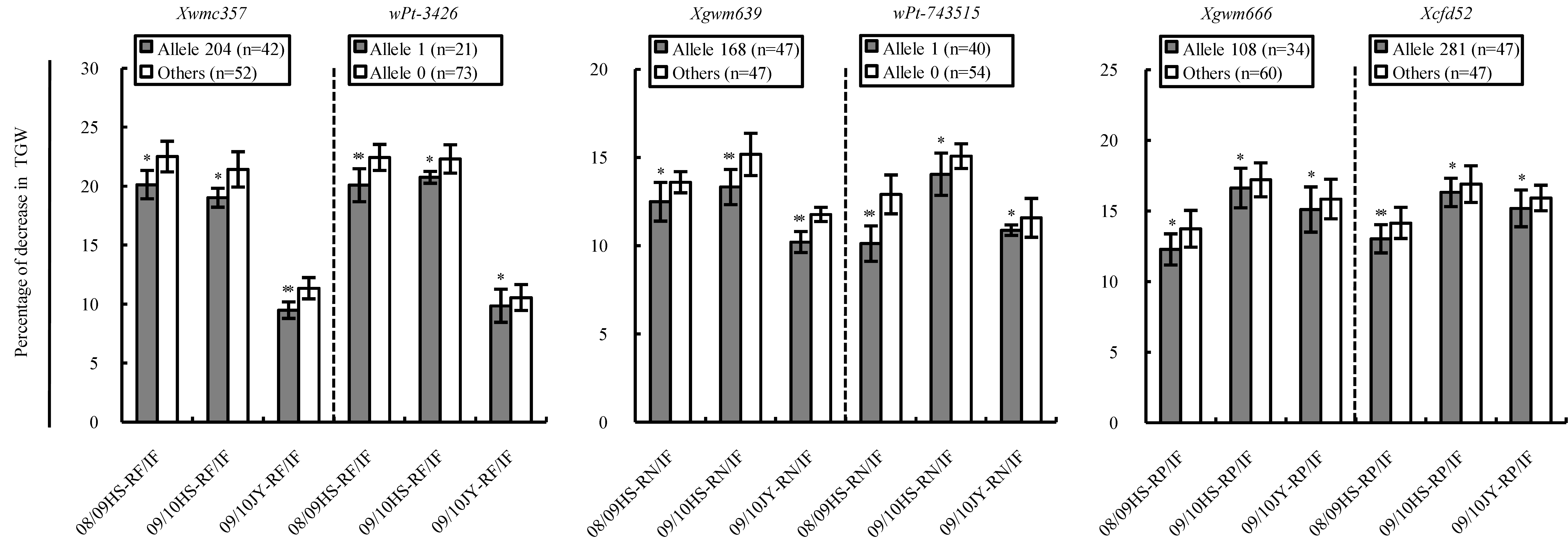

Supplement: Figure S4 — Assessment of tolerance to the decrease in thousand-grain weight (TGW) (g) conferred by the elite alleles of several associated loci under the rainfed (RF) (left panel), reduced nitrogen (RN) (middle panel), or reduced phosphorus (RP) (right panel) conditions. For the diversity arrays technology (DArT) loci (wPt-3426 and wPt-743515), the elite alleles (indicated by Allele 1) refer to the presence of their corresponding DArT sequences. For the microsatellite loci (Xwmc357, Xgwm639, Xgwm666, and Xcfd52), the elite alleles are represented by the actual size of specific amplicons (Allele 204, Allele 168, Allele 108, and Allele 281 for the four loci, respectively). “Allele 0” and “Others” are inferior alleles. The average TGWs from the RF (RN or RP) environments were compared to the corresponding values obtained under the IF conditions. Varieties carrying the elite alleles generally exhibited much less decreases in TGW than those with the inferior alleles. The number of lines (n) in each varietal group is provided in brackets. *and **indicate statistical significance at P≤0.05 and 0.01, respectively. (TIF) [file pone.0057853.s004.tif]

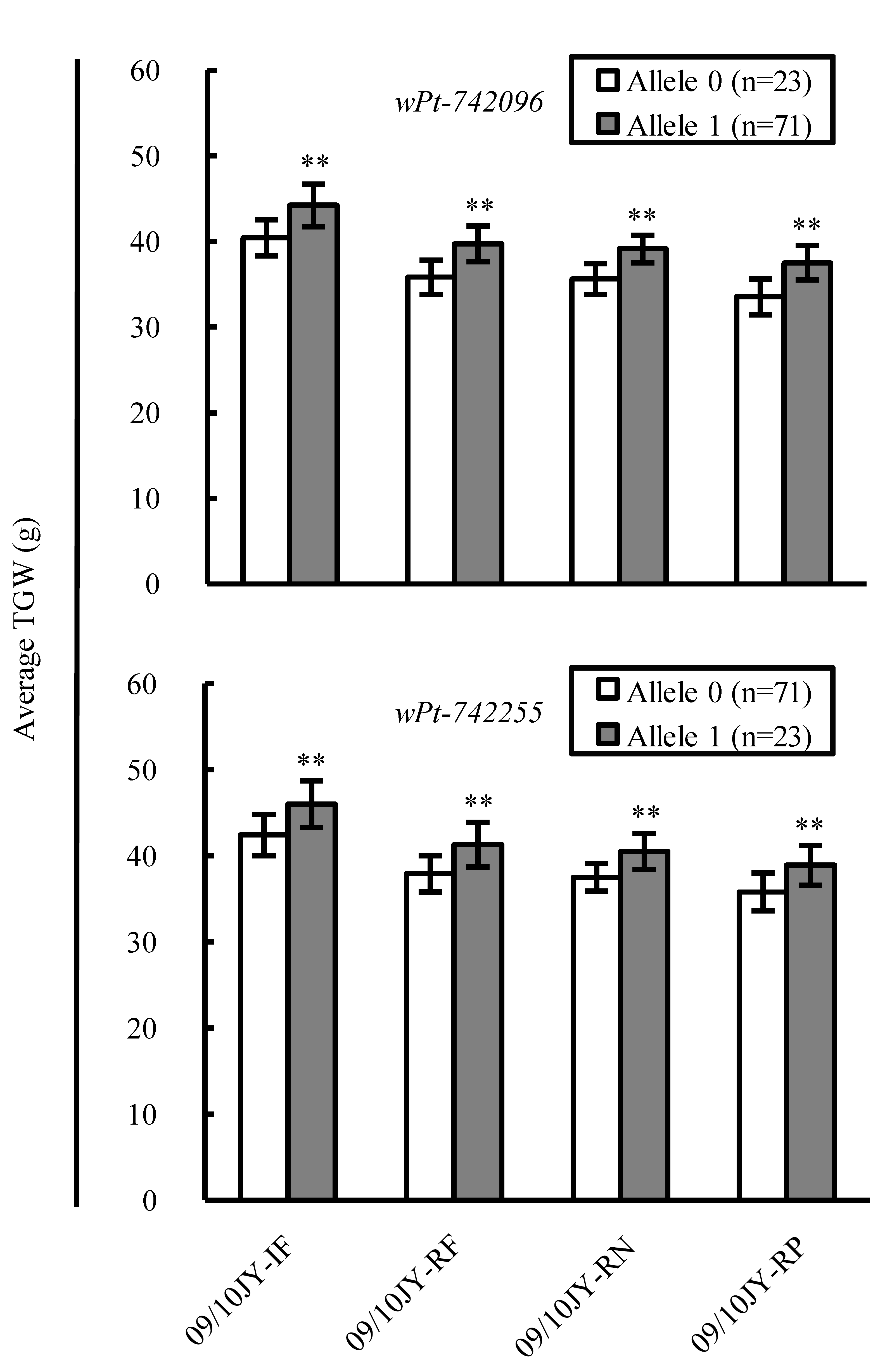

Supplement: Figure S5 — Evaluation of the phenotypic effects of wPt-742096 and wPt-742255 associated with thousand-grain weight (TGW) at the Jiyuan (JY) experimental site through marker allele-assisted genotyping. The elite and inferior alleles of the two diversity arrays technology (DArT) loci are represented by “Allele 1” and “Allele 0", respectively. Relative to the inferior alleles, the elite alleles of wPt-742096 and wPt-742255 generally had positive effects on the average TGW (g) across the four JY environments irrespective of cultivation treatment. The number of lines (n) in each varietal group is provided in brackets. *and **indicate statistical significance at P≤0.05 and 0.01, respectively. (TIF) [file pone.0057853.s005.tif]
