# Supplementary material for: Association Analysis of Genomic Loci Important for Grain Weight Control in Elite Common Wheat Varieties Cultivated with Variable Water and Fertiliser Supply
Source: PLoS One. 2013 Mar 4;8(3):e57853. doi: 10.1371/journal.pone.0057853 (PMC3587626; doi:10.1371/journal.pone.0057853)
Supplement: Table S1 — Schemes of water and fertiliser supply in the four cultivation treatments tested in this study. (DOC) [file pone.0057853.s006.doc]

**Table S1. Schemes of water and fertiliser supply in the four cultivation treatments tested in this study.**

| **Cultivation treatment** | **Irrigation (m^3^/ha)** | | | **Nitrogen fertiliser**  **(N, kg/ha)** | | **Phosphorus fertiliser (P_2_O_5_, kg/ha)** | |
| --- | --- | --- | --- | --- | --- | --- | --- |
|  | **Overwintering stage** | **Jointing stage** | **Heading stage** | **Sowing stage** | **Jointing stage** | **Sowing stage** | **Jointing stage** |
| IF | 600 | 600 | 600 | 135 | 90 | 63 | 42 |
| RF | NA | NA | NA | 135 | 90 | 63 | 42 |
| RN | 600 | 600 | 600 | 135 | NA | 63 | 42 |
| RP | 600 | 600 | 600 | 135 | 90 | 63 | NA |

NA, not applied.
