# Supplementary material for: Association Analysis of Genomic Loci Important for Grain Weight Control in Elite Common Wheat Varieties Cultivated with Variable Water and Fertiliser Supply
Source: PLoS One. 2013 Mar 4;8(3):e57853. doi: 10.1371/journal.pone.0057853 (PMC3587626; doi:10.1371/journal.pone.0057853)
Supplement: Table S2 — Differences in natural rainfall between Hengshui (HS) and Jiyuan (JY) and general performance of the 94 varieties under the irrigated and fertilised (IF), rainfed (RF), reduced nitrogen (RN), and reduced phosphorus (RP) conditions during the 08/09 and 09/10 wheat crop cycles. (DOC) [file pone.0057853.s007.doc]

**Table S2.** Differences in natural rainfall between Hengshui (HS) and Jiyuan (JY) and general performance of the 94 varieties under the irrigated and fertilised (IF), rainfed (RF), reduced nitrogen (RN), and reduced phosphorus (RP) conditions during the 08/09 and 09/10 wheat crop cycles.

|  | **HS** | | **JY** |
| --- | --- | --- | --- |
|  | **08/09 ^1^** | **09/10 ^2^** | **09/10 ^3^** |
| Rainfall (mm) | 73.6 | 82.1 | 184.4 |
| Heading date | 27-30, April (IF)  24-26, April (RF)  26-28, April (RN)  25-27, April (RP) | 30, April-02, May (IF)  26-28, April (RF)  28, April-01, May (RN)  27-30, April (RP) | 24-27, April (IF)  21-24, April (RF)  23-25, April (RN)  22-26, April (RP) |
| Harvest date | 07-10, June (IF)  04-06, June (RF)  06-08, June (RN)  05-07, June (RP) | 09-11, June (IF)  06-08, June (RF)  08-10, June (RN)  07-09, June (RP) | 05-08, June (IF)  03-05, June (RF)  06-08, June (RN)  05-07, June (RP) |
| Plant height (cm) | 57.2-100.0 (IF)  40.7-90.1 (RF)  45.2-93.5 (RN)  44.7-92.5 (RP) | 58.7-103.2 (IF)  43.8-91.0 (RF)  47.8-94.6 (RN)  47.2-93.9 (RP) | 71.5-115.2 (IF)  54.0-99.5 (RF)  60.1-107.1 (RN)  60.8-109.1 (RP) |
| Effective tiller number (m^2^) | 324-722 (IF)  246-581(RF)  255-619 (RN)  300-634 (RP) | 414-741 (IF)  254-607 (RF)  272-637 (RN)  307-652 (RP) | 437-812 (IF)  297-623 (RF)  311-669 (RN)  337-672 (RP) |

^1^The heading and harvest dates for the general wheat crop in 08/09 cycle in HS were 26 April to 1 May and 5-11 June, respectively;

^2^The heading and harvest dates for the general wheat crop in 09/10 cycle in HS were 28 April to 3 May and 7-13 June, respectively;

^3^The heading and harvest dates for the general wheat crop in 09/10 cycle in JY were 22-28 April and 03-09 June, respectively.
