# Supplementary material for: Association Analysis of Genomic Loci Important for Grain Weight Control in Elite Common Wheat Varieties Cultivated with Variable Water and Fertiliser Supply
Source: PLoS One. 2013 Mar 4;8(3):e57853. doi: 10.1371/journal.pone.0057853 (PMC3587626; doi:10.1371/journal.pone.0057853)
Supplement: Table S5 — Positive influence of wPt-2464 elite allele on grain length (GL, mm) and grain width (GW, mm) in the irrigated and fertilised (IF), rainfed (RF), reduced nitrogen (RN), and reduced phosphorus (RP) environments during the 08/09 wheat crop cycle in Hengshui (HS). (DOC) [file pone.0057853.s010.doc]

Table S5. Positive influence of *wPt-2464* elite allele on grain length (GL, mm) and grain width (GW, mm) in the irrigated and fertilised (IF), rainfed (RF), reduced nitrogen (RN), and reduced phosphorus (RP) environments during the 08/09 wheat crop cycle in Hengshui (HS).

| **Locus** |  | **IF (HS)** | | **RF (HS)** | | **RN (HS)** | | **RP (HS)** | |
| --- | --- | --- | --- | --- | --- | --- | --- | --- | --- |
|  |  | **GL** | **GW** | **GL** | **GW** | **GL** | **GW** | **GL** | **GW** |
| *wPt-2464* | Allele 1 (n = 25 ) | 6.82 ± 0.20^**^ | 3.49 ± 0.14^*^ | 6.40 ± 0.25^**^ | 3.21 ± 0.16^*^ | 6.47 ± 0.27^**^ | 3.32 ± 0.15^*^ | 6.50 ± 0.24^**^ | 3.33 ± 0.15^**^ |
|  | Allele 0 (n = 69 ) | 6.63 ± 0.17 | 3.41 ± 0.16 | 6.30 ± 0.27 | 3.13 ± 0.14 | 6.28 ± 0.27 | 3.25 ± 0.12 | 6.29 ± 0.26 | 3.23 ± 0.14 |

Allele 1 was elite; “n” denotes the number of varieties carrying the given allele; Statistical comparison was made between the averaged measurements of the two allele types; * and ** indicate *P* ≤ 0.05 (significant) or 0.01 (highly significant).
