# Supplementary material for: Association Analysis of Genomic Loci Important for Grain Weight Control in Elite Common Wheat Varieties Cultivated with Variable Water and Fertiliser Supply
Source: PLoS One. 2013 Mar 4;8(3):e57853. doi: 10.1371/journal.pone.0057853 (PMC3587626; doi:10.1371/journal.pone.0057853)
Supplement: Table S6 — Positive influence of wPt-742096 and wPt-742255 elite alleles on grain length (GL, mm) and grain width (GW, mm) in the irrigated and fertilised (IF), rainfed (RF), reduced nitrogen (RN), and reduced phosphorus (RP) environments during the 09/10 wheat crop cycle in Jiyuan (JY). (DOC) [file pone.0057853.s011.doc]

**Table S6.** Positive influence of *wPt-742096* and *wPt-742255* elite alleles on grain length (GL, mm) and grain width (GW, mm) in the irrigated and fertilised (IF), rainfed (RF), reduced nitrogen (RN), and reduced phosphorus (RP) environments during the 09/10 wheat crop cycle in Jiyuan (JY).

| **Locus** |  | **IF (JY)** | | **RF (JY)** | | **RN (JY)** | | **RP (JY)** | |
| --- | --- | --- | --- | --- | --- | --- | --- | --- | --- |
|  |  | **GL** | **GW** | **GL** | **GW** | **GL** | **GW** | **GL** | **GW** |
| *wPt-742096* | Allele 1 (n = 71) | 6.47 ± 0.27^*^ | 3.38 ± 0.16^**^ | 6.36 ± 0.28 | 3.30 ± 0.14^**^ | 6.38 ± 0.27^*^ | 3.37 ± 0.15^**^ | 6.40 ± 0.26^*^ | 3.33 ± 0.16^**^ |
|  | Allele 0 (n = 23 ) | 6.41 ± 0.26 | 3.25 ± 0.17 | 6.28 ± 0.21 | 3.16 ± 0.15 | 6.31 ± 0.24 | 3.26 ± 0.16 | 6.33 ± 0.25 | 3.21 ± 0.15 |
| *wPt-742255* | Allele 1 (n = 23) | 6.58 ± 0.28^**^ | 3.40 ± 0.16^*^ | 6.47± 0.29^*^ | 3.33 ± 0.14^**^ | 6.53 ± 0.27^**^ | 3.45 ± 0.17^**^ | 6.54 ± 0.25^**^ | 3.37 ± 0.15^*^ |
|  | Allele 0 (n = 71 ) | 6.41 ± 0.26 | 3.33 ± 0.18 | 6.29 ± 0.24 | 3.24 ± 0.15 | 6.32 ± 0.24 | 3.31± 0.15 | 6.34 ± 0.24 | 3.28 ± 0.16 |

Allele 1 was elite; “n” denotes the number of varieties carrying the given allele; Statistical comparison was made between the averaged measurements of the two allele types; * and ** indicate *P* ≤ 0.05 (significant) or 0.01 (highly significant).
