# Supplementary material for: Association Analysis of Genomic Loci Important for Grain Weight Control in Elite Common Wheat Varieties Cultivated with Variable Water and Fertiliser Supply
Source: PLoS One. 2013 Mar 4;8(3):e57853. doi: 10.1371/journal.pone.0057853 (PMC3587626; doi:10.1371/journal.pone.0057853)
Supplement: Table S7 — Comparisons of the 15 significantly associated loci found in this work with previously identified loci affecting grain, yield, and related traits. (DOC) [file pone.0057853.s012.doc]

**Table S7.** Comparisons of the 17 significantly associated loci found in this work with previously identified loci affecting grain, yield, and related traits.

| **Locus, chromosomal** | | | **Type of association^1^** | **Previously identified loci affecting grain (weight, length and width), yield, and related traits** | |
| --- | --- | --- | --- | --- | --- |
| **location, and genetic position (cM)** | | |  | **Correspondence based on the sharing of common marker and the similarity in chromosomal location and genetic position** | **Correspondence based on only the similarity in chromosomal location and genetic position** |
| *Xgwm299* | 3BL | 87.78 | EI | 1)      QTL for grain weight [*QTgw.ipk-3B.2*, *Xgwm299*] [85] | 1)      Locus associated with grain weight [*Xgwm547*] [8] |
|  |  |  |  | 2)      Meta-QTL for yield and related traits [*MQTL29*, *bg131*-*Xgwm299*] [86] |  |
|  |  |  |  | 3)      QTL for grain weight [*Xgwm299*] [87] |  |
| *Xpsp3152* | 6AL | 80.66 |  | 1)      Meta-QTL for grain length [*MQTL_8*, *Xpsp3029*-*Xgwm570*-*Xpsp3071*- | 1)      *TaGW2* for grain weight [7] |
|  |  |  |  | *Xpsp3152*-*Xwmc553*-*Xwmc179*] [39] |  |
| *Xwmc17* | 7AL | 89.2 |  | 1)      Meta-QTL for yield and related traits [*MQTL49*, *Xwmc83*-*Xwmc17*] [86] | 1)      QTL for grain weight and other yield related traits [*Xwmc83*] [22] |
|  |  |  |  | 2)      Locus associated with grain weight [*Xwmc17*] [8] | 2)      QTL for grain weight under low phosphorus conditions [*Xwmc488*] [32] |
| *wPt-6965* | 3BS | 10.28 |  | Not found | 1)      QTL for yield [*Xgwm493*] [13] |
|  |  |  |  |  | 2)      QTL for yield [*Xbarc133*-*Xgwm493*] [88] |
|  |  |  |  |  | 3)      QTL for grain weight, grain filling rate and related traits [*Xbarc113*-*Xgwm533*] [89] |
| *Xgwm637* | 4AL | 93.86 | ED (IF) | 1)      QTL for yield and related traits [*MQTL32*, *Xgwm637*-*Xgwm2228*] [86] | 1)      QTL for green leaf duration after heading [*Xbarc170* - *Xgpw3238*] [91] |
|  |  |  |  | 2)      QTL for yield and related traits [*Xgwm637*-*wPt-6515*-*wPt-7558*] [90] |  |
| *Xbarc1* | 5AS | 33.04 |  | Not found | 1)      QTL for grain length [*Xcfa2250*-*Xbarc141*] [36] |
|  |  |  |  |  | 2)      QTL for grain weight [*Xgwm186*] [89] |
|  |  |  |  |  | 3)      Meta-QTL for yield and related traits [*MQTL40*, *Xbcd1108*-*Xgwm186*] [86] |
| *Xpsp3071* | 6AL | 95.09 |  | 1)      QTL for grain weight and yield [*Xwmc32*-*Xpsp3071*] [92] | 1)      Locus associated with grain weight [*Xbarc171*] [50] |
|  |  |  |  | 2)      QTL for grain weight [*Xcwm306*-*Xpsp3071*, *Xpsp3071*-*Xgwm570*] [32] |  |
|  |  |  |  | 3)      Meta-QTL for grain length [*MQTL_8*, *Xpsp3029*-*Xgwm570*-*Xpsp3071*- *Xpsp3152*-*Xwmc553*-*Xwmc179*] [39] |  |
| *Xbarc235* | 7DL | 160.57 |  | Not found | 1)      QTL for grain weight [*Xgwm428*] [15] |
|  |  |  |  |  | 2)      QTL for yield [*Xgwm428-Xgwm37*] [17] |
|  |  |  |  |  | 3)      Meta-QTL for yield and related traits [*MQTL55*, *Xmwg975*-*Xfba204*] [86] |
|  |  |  |  |  |  |
| *Xwmc357* | 5DL | 80.63 | ED (RF) | Not found | 1)      *Vrn-D1* [70] |
| *Xgwm639* | 5DL | 43.67 | ED (RN) | 1)      QTL for straw nitrogen content under high nitrogen conditions [*Xgwm639*] [30] | 1)      QTL for grain protein yield under low nitrogen conditions [*Xcfd8*] [30] |
|  |  |  |  | 2)      QTL for grain weight [*Xcfd8-Xgwm639*] and glutamine synthetase activity [*Xgwm639-Xcfd12*] [31] | 2)      QTL for root nitrogen content and total nitrogen content per plant [*Xgwm182*] [34] |
| *Xwmc486* | 6BS | 6.05 |  | 1)      Locus associated with heading date and peduncle length [*Xwmc486*] [50] | Not found |
| *Xgwm666* | 5AL | 73.41 | ED (RP) | 1)      QTL for shoot phosphorus uptake per plant and shoot phosphorus utilization efficiency [*Xgwm666*-*Xgwm271*] [94] | 1)      *Vrn-A1* [94] |
|  |  |  |  | 2)      Meta-QTL for grain length and width [*MQTL_6*, *Xwmc492*-*Xgwm666*] [39] |  |
|  |  |  |  | 3)      Meta-QTL for yield related traits [*MQTL41*, *Xwmc327*-*Xgwm666*] [86] |  |
| *Xcfd52* | 5DL | 64.79 |  | Not found | Not found |
| *wPt-5432* | 3BS | 36.9 | SD (HS) | Not found | 1)      Locus associated with grain weight [*Xgwm156*] [8] |
| *wPt-2464* | 3DS | 14.41 |  | Not found | Not found |

^1^ED, EI and SD refer to environment-dependent, environment-independent, and site-dependent, respectively; IF, RF, RN, and RP indicate irrigated and fertilised, rainfed, reduced nitrogen, and reduced phosphorus treatments, respectively; HS, Hengshui.
